# ERAP, KIR, and HLA-C Profile in Recurrent Implantation Failure

**DOI:** 10.3389/fimmu.2021.755624

**Published:** 2021-10-22

**Authors:** Karolina Piekarska, Paweł Radwan, Agnieszka Tarnowska, Andrzej Wiśniewski, Michał Radwan, Jacek R. Wilczyński, Andrzej Malinowski, Izabela Nowak

**Affiliations:** ^1^ Laboratory of Immunogenetics and Tissue Immunology, Department of Clinical Immunology, Ludwik Hirszfeld Institute of Immunology and Experimental Therapy, Polish Academy of Sciences, Wrocław, Poland; ^2^ Department of Reproductive Medicine, Gameta Hospital, Rzgów, Poland; ^3^ Faculty of Health Sciences, The Mazovian State University in Płock, Płock, Poland; ^4^ Department of Surgical and Oncological Gynecology, Medical University of Łódź, Łódź, Poland; ^5^ Department of Surgical, Endoscopic and Oncologic Gynecology, Polish Mothers’ Memorial Hospital—Research Institute, Łódź, Poland; ^6^ Medical Centre Gynemed, Łódź, Poland

**Keywords:** ERAP, KIR, HLA-C, polymorphism, IVF, RIF

## Abstract

The mother’s uterine immune system is dominated by uterine natural killer (NK) cells during the first trimester of pregnancy. These cells express killer cell immunoglobulin-like receptors (KIRs) of inhibitory or activating function. Invading extravillous trophoblast cells express HLA-C molecules, and both maternal and paternal HLA-C allotypes are presented to KIRs. Endoplasmic reticulum aminopeptidase 1 (ERAP1) and 2 (ERAP2) shape the HLA class I immunopeptidome. The ERAPs remove N-terminal residues from antigenic precursor peptides and generate optimal-length peptides to fit into the HLA class I groove. The inability to form the correct HLA class I complexes with the appropriate peptides may result in a lack of immune response by NK cells. The aim of this study was to investigate the role of *ERAP1* and *ERAP2* polymorphisms in the context of *KIR* and *HLA-C* genes in recurrent implantation failure (RIF). In addition, for the first time, we showed the results of ERAP1 and ERAP2 secretion into the peripheral blood of patients and fertile women. We tested a total of 881 women. Four hundred ninety-six females were patients who, together with their partners, participated in *in vitro* fertilization (IVF). A group of 385 fertile women constituted the control group. Women positive for *KIR* genes in the Tel AA region and *HLA-C2C2* were more prevalent in the RIF group than in fertile women (p/p_corr._ = 0.004/0.012, OR = 2.321). Of the *ERAP* polymorphisms studied, two of them (rs26653 and rs26618) appear to affect RIF susceptibility in HLA-C2-positive patients. Moreover, fertile women who gave birth in the past secreted significantly more ERAP1 than IVF women and control pregnant women (p < 0.0001 and p = 0.0005, respectively). In the case of ERAP2, the opposite result was observed; i.e., fertile women secreted far less ERAP2 than IVF patients (p = 0.0098). Patients who became pregnant after *in vitro* fertilization embryo transfer (IVF-ET) released far less ERAP2 than patients who miscarried (p = 0.0032). Receiver operating characteristic (ROC) analyses indicate a value of about 2.9 ng/ml of ERAP2 as a point of differentiation between patients who miscarried and those who gave birth to a healthy child. Our study indicates that both ERAP1 and ERAP2 may be involved in processes related to reproduction.

## Introduction

Despite the substantial progress in assisted reproductive technologies (ARTs), a high percentage of embryos (50%) are lost at once after implantation or shortly after as miscarriage (1). However, the most stressful problem from the economic and psychological point of view for embryologists and infertile couples is recurrent implantation failure (RIF), which affects 10%–15% of couples having undergone several *in vitro* fertilization embryo transfers (IVF-ETs). RIF is commonly defined as a failure to achieve a pregnancy after three subsequent IVF cycles, in which four good-quality embryos were transferred in women under the age of 40 years (2–4).

Successful maternal tolerance to the semi-allogeneic fetus is a complicated process. The fetal trophoblast cells come into direct contact with the mother’s immune system in the uterus. They constitute the layer that surrounds the blastocyst (5, 6). The extravillous trophoblast (EVT) cells invade the decidua at implantation and during placentation to transform the arteries and establish blood supply to the placenta. Insufficient invasion of trophoblasts and vascular alteration in the decidua are thought to be the primary defect in recurrent miscarriage, preeclampsia (PE), and fetal growth restriction (7, 8).

The mother’s uterine immune system is dominated by uterine natural killer (NK) cells, CD56^bright^CD16−, the most common leukocyte population during the first trimester of human pregnancy (8–10). Decidual NK cells (dNKs) are poorly cytolytic, and they release cytokines/chemokines and growth factors that induce trophoblast invasion, tissue remodeling, embryonic development, and placentation (8). These cells express killer cell immunoglobulin-like receptors (KIRs). In general, KIR nomenclature is based on the arrangement of the extracellular immunoglobulin-like (Ig) domains (2D or 3D indicates the Ig domain number) and the length of the intracytoplasmic tail (S and L indicate a short tail and a long tail, respectively). The length of the cytoplasmic fragment relates to the type of NK function mediated by particular KIRs. Activating receptors have a short cytoplasmic fragment with the immunoreceptor tyrosine-based activation motif (ITAM), and are marked with the letter S (short). In turn, inhibitory receptors have a long cytoplasmic tail with an immunoreceptor tyrosine-based inhibitory motif (ITIM) and are marked with the letter L (long). The KIR2DL4 receptor is an exception, however, as it may conduct both activation and inhibitory signals (11–13). NK cell function depends on the balance between those activating and inhibitory receptors (14).

KIRs are encoded by a family of genes on the leukocyte receptor complex on chromosome 19q13.4 (15). *KIR* genes exhibit extensive haplotypic polymorphism. Individuals differ in both the number and kind (activating *vs.* inhibitory) of *KIR* genes. *KIR* genes can be organized into two haplotypes: A and B. Both haplotypes consist of framework genes: *KIR3DL3* at the centromeric end, *KIR3DL2* at the telomeric end, and *KIR3DP1* and *KIR2DL4* in the middle of KIR gene cluster. Group A haplotypes possess *KIR2DL1*, *KIR2DL3*, *KIR3DL1*, *KIR2DS4*, and *KIR2DP1* and exert inhibition of cell function. Group B haplotypes differ in the number and combination of *KIR* genes. They may possess one or more of *KIR2DL1*, *KIR2DL2*, *KIR2DL5A/B*, *KIR2DS1*, *KIR2DS2*, *KIR2DS3*, *KIR2DS5*, and *KIR3DS1* genes. An individual with two copies of haplotype A is considered as AA genotype, while an individual with haplotype B genes is considered as the Bx genotype (AB+BB). Thus, the AA genotype is a set of genes for the largest number of inhibitory receptors (16–18). The intensity of inhibition decreases with an increase in the number and expression of genes for activating KIRs (19). All necessary information regarding KIRs and genes is available on the website: (https://www.ebi.ac.uk/ipd/kir/) (accessed December 16, 2020).

The ligands for the inhibitory and activating KIRs constitute HLA-A, HLA-B, HLA-C, and HLA-G allotypes (20). Invading EVTs express HLA-C molecules in the decidua basalis, and both maternal and paternal HLA-C allotypes are presented to KIRs (7). The *HLA-C* gene is polymorphic. Generally, *HLA-C* alleles may occur in two allotypes: C1 and C2 based on the presence of asparagine or lysine at position 80 of the HLA-C α-domain. HLA-C1 allotypes bind inhibitory KIR2DL2/3, while HLA-C2 allotypes bind KIR2DL1 and KIR2DS1. However, the interaction of the former is weaker than in KIR2DL1-HLA-C2 (20–25). Therefore, the balance of *KIR* genotypes in a given patient and the HLA-C exposure in a given pregnancy may affect trophoblast invasion, vascular remodeling, and the initiation of normal placentation.

Endoplasmic reticulum (ER) aminopeptidase 1 (ERAP1) and 2 (ERAP2) critically shape the HLA class I immunopeptidome. The ERAPs remove N-terminal residues from antigenic precursor peptides and generate optimal-length peptides (i.e., 8–10-mers) to fit into the HLA class I groove (26). ERAPs can also destroy the putative HLA class I ligands by reducing the length of antigenic peptides below the threshold of 8–10 amino acids when they are overactive (26). ERAP1/ERAP2 protein expression is detected in many tissues and is induced by type I and type II interferon (IFN) and tumor necrosis factor-alpha (TNF-α) (27–30).

Like the *KIR* and *HLA-C* genes, *ERAP* genes are also polymorphic with strong linkage disequilibrium (LD) across the chromosome 5q15 locus, and many functional variants appear to affect their enzymatic activity or the expression level or both (27, 31). Polymorphic amino acids are located near the catalytic site (residue 349—rs2287987), in the peptide binding site (residue 730—rs27044), or in a location that can affect the conformational changes associated with enzymatic activity (residue 528—rs30187). *In vitro* studies have demonstrated that 528R has lower enzymatic activity and protein expression in contrast to 528 K (32–34). At the domain junction, there is also rs26653 (R127P), which could alter the conformation between open and closed forms and therefore impact specificity and enzymatic activity (35). In turn, rs26618 (I276M) affects efficiency of a precursor peptide trimming for the HLA-C*05-bound epitope (36). Polymorphism rs6861666 A>G appears in 100% LD with rs75862629, which influences the expression level of ERAP2 and ERAP1 (37). The polymorphism rs2549782 of *ERAP2* coding for the K392N change affects ERAP2 activity. The N392 allele has strong LD with single-nucleotide polymorphism (SNP) rs2248374 G>A, a polymorphism that favors nonsense mediated RNA decay and impairs protein expression (38). Because both alleles of rs2248374 occur with a similar frequency in the population due to balancing selection, about 25% of individuals fail to express ERAP2 (39). Therefore, these polymorphisms may influence ERAP1 and ERAP2 activities in multiple ways (33, 40).

The inability to form the correct HLA class I complexes with the appropriate peptides may result in a lack of immune response by CD8+ lymphocytes and NK cells. Therefore, *ERAP1* and *ERAP2* polymorphisms may significantly contribute to the interactions between KIR-HLA-C at the maternal–fetal interface.

The aim of this study was to investigate the role of *ERAP1* and *ERAP2* polymorphisms in the context of *KIR* and *HLA-C* genes in women suffering RIF in the Polish population. However, due to a large number of research results, this article applies only to women participating in IVF. Potential interactions between a mother’s ERAP and KIR and her partner’s HLA-C will be the subject of another publication.

In addition, for the first time, we want to show the results of studies on the secretion of ERAP1 and ERAP2 into the peripheral blood of IVF patients and fertile women, which indicate the dependence of pregnancy success on the secretion of ERAP1 and ERAP2.

## Material and Methods

### Study Design

In our research, we tested a total of 881 women. Four hundred ninety-six females were patients who, together with their partners, participated in IVF, with a total of 1,859 assisted reproductive cycles. A group of 385 fertile women constituted the control group. Patients were qualified at the Gameta Assisted Reproduction Clinic in Rzgów, a center certified by the European Society of Human Reproduction and Embryology (ESHRE ART Centre Certification for good clinical practice). Patients were also recruited from the Department of Surgical, Endoscopic and Oncologic Gynecology and the Department of Gynecology and Gynecologic Oncology, Polish Mothers’ Memorial Hospital—Research Institute in Łódź and Medical Centre Gynemed in Łódź. Patients were included in the studies from 2015 to 2020. The control group was qualified mainly from the 1st Department of Obstetrics and Gynecology, Medical University of Warsaw, in the years 2006–2014. The control group was also qualified at the Institute of Immunology and Experimental Therapy of the Polish Academy of Sciences in 2018–2020. These women and their partners had at least one healthy child from natural conception. All tested couples were of Polish origin. The clinical characteristics of couples who participated in IVF and the control group are presented in **Table 1**. Both IVF patients and their partners differed significantly in mean age from fertile couples (p = 0.0001 and p < 0.0001, respectively). Detailed information on the preparation of patients for IVF [ovarian stimulation, fertilization procedure, endometrial preparation, and frozen ET (FET)] has been described earlier by Nowak et al. (41).

**Table 1:** Clinical characteristics of couples participating in IVF-ET and fertile control.

Aspect		ALL IVF	RIF	SIVF	Unclassified	Fertile control
		N = 496	N = 283	N = 161	N = 52	N = 385
Age of woman	Mean ± SD	**33.67^a^** ± 4.14	**34.51^b,c^** ± 4.11	32.11 ± 3.82	34.09 ± 3.91	32.81 ± 5.98
	Range	22–46	23–46	22–41	25–45	19–68
Age of partner	Mean ± SD	**35.51^d^** ± 4.89	**36.27^e,f^** ± 4.85	34.32 ± 4.72	36.89 ± 4.89	34.15 ± 6.3
	Range	24–53	25–53	24–53	28–51	25–70
Indications for IVF-ET	Only male factor	145 (29.23)	78 (27.57)	58 (36.02)	9 (17.31)	N/A
	Only female factor	131 (26.41)	70 (24.73)	45 (27.95)	16 (30.77)	N/A
	Both factors	75 (15.13)	49 (17.31)	19 (11.80)	7 (13.46)	N/A
	Unknown factor	145 (29.23)	86 (30.39)	39 (24.23)	20 (38.46)	N/A
Number of IVF-ET	Mean ± SD	3.37 ± 2.05	4.64 ± 1.76	1.63 ± 0.71	1.62 ± 0.68	N/A
	Range	1–15	3–15	1–3	1–3	N/A
Number of embryos	Mean ± SD	3.81 ± 2.55	5.33 ± 2.29	1.69 ± 0.79	1.76 ± 0.65	N/A
	Range	1–19	3–19	1–5	1–3	N/A

Values in bold indicate significant differences. Values in parentheses are in percentages.

IVF-ET, in vitro fertilization embryo transfer; RIF, recurrent implantation failure; SIVF, successful pregnancy after IVF-ET; SD, standard deviation; p, probability.

ALL vs. Fertile: women: ^a^p = 0.0001, partner: ^d^p < 0.0001; RIF vs. Fertile: women: ^b^p < 0.0001, partner: ^e^p < 0.0001; RIF vs. SIVF: women: ^c^p < 0.0001, partner: ^f^p = 0.0001.

### Luteal Phase Support and Steroid Treatment

Patients undergoing standard IVF procedure were instructed to routinely take 5 mg of prednisone (Encorton, Adamed, Poland) once a day in the morning orally, starting from the day of ET (ET/FET). Patients with RIF and elevated TNF-α level received steroids in higher doses (10–20 mg) for 2–3 weeks before ET and up to 8 weeks if pregnancy developed after transfer. In order to supplement the luteal phase, patients were intravaginally administered 2 × 200 mg micronized progesterone (Luteina, Adamed, Poland) and oral dydrogesterone 3 × 10 mg (Duphaston, Solvay Pharmaceuticals, Netherlands) until 12 weeks of gestation.

### DNA Preparation and Genotyping

Genomic DNA was isolated from venous blood using the Invisorb Spin Blood Midi Kit (Invitek, Germany) or QIAamp DNA Mini Kit (Qiagen, Germany) according to the manufacturer’s instructions. *KIR*s were genotyped using KIR Ready Gene kits (Inno-train Diagnostics, Germany) following the manufacturer’s instructions [for details, see reference (42) and **Table 2**)] or multiplex PCR described elsewhere (43, 44). Our *KIR* typing was validated three times per year by the International KIR Exchange program organized by the Immunogenetics Center of the University of California, Los Angeles. *KIR* AA genotype was estimated by the presence of *KIR2DL1*, *KIR2DL3*, *KIR2DS4*, and *KIR3DL1* and the absence of *KIR2DL5*, *KIR2DS1*, *KIR2DS2*, *KIR2DS3*, *KIR2DS5*, and *KIR3DS1*, which may be found in the *KIR* Bx genotype. *KIR*s were also divided according to presence in the centromeric or telomeric part of *KIR* gene cluster: CenA-KIR2DL3, CenB-KIR2DL2 and KIR2DS2, TelA-KIR3DL1 and KIR2DS4, and TelB-KIR2DS1 and KIR3DS1 (45). The genes of the Cen AA/Tel AA combination constitute the AA genotype, while all other combinations constitute the Bx genotype. *HLA-C1* and *C2* allotypes were detected by a PCR-SSP method described in detail elsewhere (46). *ERAP* genotyping was performed using the TaqMan SNP Genotyping Assay (Applied Biosystems, USA) as described in more detail previously (45). Characteristics of the *ERAP* SNPs examined in this study are shown in **Table 3**.

**Table 2:** KIR allele specificity.

KIR gene type	Allele specificity
2DL1	*001–022, 024–026N
2DL2	*001–003, 005, 007–010, 012/2DP1*012
2DL3	*001–003, (004), 005–009, 011–017, 019–024/2DS4*013
2DL4 norm	*0001–006, 010, 012, 014–016, 018, 021–026
2DL4 deleted	*007–009, 011, 013, 017, 019, 020, 027
2DL5 all	A*all (001, 005, 012, 014, 015), B*all (002–004, 006–011, 013, 016–018)
2DL5 (group 1)	A*001, 012, 014, 015, B*003, 004, 006–008, 011, 013, 018
2DL5 (group 2)	A*005, B*002, 009, 010, 016, 017
2DL5 expressed	A*001, 005:01:01, 005:01:03, 005:01:04, 012, (014, 015)/3DP1*004, (002, 011–014)
2DL5 null	B*002, 004, 006:01, 006:03, 007–011, 013 (0070102, 00803, 01303, 016–018)/3DP1*001, 007, 009:01, (011–014)
2DS1	*002, 003, 005, 006
2DS2	*001–008
2DS3	*001–007
2DS4 norm	*001, 011, 014, 015
2DS4 (del-22bp)	*003–010, 012, 013
2DS5	*001–012
3DL1	*001–008, 015–026, (028), 029–041, 043–056, 059–076/3DS1*014
3DL2	*001–017, 019–063
3DL3	*001–057
3DS1	*010–058
2DP1	*001–014
3DP1 norm	*003, 005, 006, 008, 010, 013, 014
3DP1 variant	*001, 002, 004, 007, 009, 011, 012

**Table 3:** Characteristics of the *ERAP* SNPs examined in this study.

Locus	Gene	SNP	SNP variation	Protein variation	Potential effect	Assay ID
5q15	*ERAP1*	rs26653	G>C	P127R	Enzymatic activity, expression level (35)	C:794818_30
5q15	*ERAP1*	rs2287987	T>C	M349V	Interactions with the substrate (35)	C:3056893_20
5q15	*ERAP1*	rs30187	C>T	R528K	Enzymatic activity, expression level (34)	C:3056885_10
5q15	*ERAP1*	rs27044	C>G	E730Q	Enzymatic activity, substrate length preference (35)	C:3056870_10
5q15	*ERAP1*	rs26618	T>C	I276M	Affects efficiency of a precursor peptide trimming for the HLA-C*05-bound epitope (36)	C:3056894_10
5q15	*ERAP1/ERAP2*	rs6861666	A>G	–	rs6861666 in 100% LD with rs75862629 which influences the expression level of ERAP2 and ERAP1 (37)	C:29091789_20
5q15	*ERAP2*	rs2248374	A>G	–	Lack of expression of functional forms of the enzyme (38, 39)	C:25649529_10

LD, linkage disequilibrium; SNP, single-nucleotide polymorphism.

### ERAP Measurement

We measured ERAP1 in 121 plasma samples collected before IVF-ET and 108 plasma samples after IVF-ET during testing of the beta-subunit of human chorionic gonadotropin. However, the concentration of ERAP2 was measured in 243 samples taken before IVF-ET and 192 samples taken from patients after IVF-ET. Plasma levels of ERAP1 and ERAP2 were also tested in 40 samples from fertile women who had previously given birth to healthy children from natural conception and 27 who were pregnant at the time of plasma collection.

Plasma samples were stored at −80°C until the time of assay. The concentration of ERAP1 and ERAP2 (ng/ml) in plasma of patients was tested with a sandwich ELISA kit following the manufacturer’s protocols (Wuhan EIAab Science Co., China). Standard curve measured the concentration of ERAP1 from 0.15 to 10.0 ng/ml and ERAP2 from 0.31 to 20.0 ng/ml. The limit of ERAP1 detection in this test was 0.085 ng/ml, while ERAP2 is less than 0.16 ng/ml.

### Statistical Analysis

For the analysis of *KIR*, *HLA-C*, and *ERAP* genotype frequencies, we used the two-tailed Fisher’s exact test (R software). The Hardy–Weinberg equilibrium was estimated using the chi-square test with one degree of freedom. All tested genotype frequencies were in the Hardy–Weinberg equilibrium except for the *ERAP1* rs2287987 polymorphism in the control group. A p-value <0.05 was considered significant. The odds ratio (OR) and its 95% confidence interval (95% CI) were computed as the measure of effect size. For multiple comparison tests, Bonferroni correction was done. Haplotypes were generated by FAMHAP19 software (http://famhap.meb.uni-bonn.de).

Statistical analyses concerning ERAP1 and ERAP2 concentration in the plasma of patients before and after ET were performed using the Mann–Whitney test (GraphPad Prism 5 software). To identify a cut-off level of ERAP suggestive of likelihood of miscarriage, receiver operating characteristic (ROC) curve analysis was performed (GraphPad Prism 5 software).

### Ethical Approval

Experimental protocols were approved by Local Ethics Committee (on agreement of Polish Mothers’ Memorial Hospital—Research Institute in Łódź), and informed consent was obtained from all individual participants included in the study.

## Results

### Comparison of *KIR*, *ERAP*, and *HLA-C* Genes and Haplotype Frequencies in *In Vitro* Fertilization Patients and Fertile Control

We found no statistically significant differences in the frequencies of both single *KIR* genes, AA and Bx genotypes, and *KIR* divided into centromeric and telomeric regions between IVF patients and fertile control. Also, the frequencies of *HLA-C* allotypes did not differ in the tested groups (**Supplementary Table 1**). In the case of *ERAP* genes, we observed weak differences for rs27044 C>G and rs26618 C>T when we compared the RIF group with the fertile control group (**Supplementary Table 2**). Moreover, we estimated 27 different *ERAP1/ERAP2* haplotypes. For three haplotypes, we found significant differences between the studied groups, albeit weak and losing significance after Bonferroni correction (**Supplementary Table 3**). Potential interactions were found between associated *KIR* and *ERAP* genes and *HLA-C* allotypes.

When we analyzed the potential interactions between the studied genes, we obtained interesting results that are summarized in **Table 4** and in detail presented in **Supplementary Tables 4**–**15**. Due to the fact that we made a large number of comparisons, we applied Bonferroni corrections. The summary in **Table 4** includes only those analyses that were still statistically significant after the correction.

**Table 4:** Summarized effect of *ERAP, HLA-C*, and *KIR* combined polymorphisms on the susceptibility to infertility and recurrent implantation failure.

ERAP, HLA-C, KIR combination	Associated combination	Compared groups	p	p_corr._	OR	95% CI	Effect	Table
KIR/HLA-C	TelAA/C2C2	ALL *vs.* Fertile	0.009	0.026	2.020	1.16–3.61	↑	Suppl.4
	TelAA/C2C2	RIF *vs.* Fertile	0.004	0.012	2.321	1.26–4.35	↑	Suppl.4
ERAP1 rs26653 G>C/HLA-C	GG/C2C2	ALL *vs.* Fertile	0.002	0.022	0.343	0.16–0.72	↓	Suppl.5
	GG/C2C2	RIF *vs.* Fertile	0.001	0.005	0.252	0.10–0.59	↓	Suppl.5
	CG/C2C2	RIF *vs.* Fertile	0.002	0.016	3.661	1.56–8.89	↑	Suppl.5
ERAP1 rs26653 G>C/KIR	GG/telBB	RIF *vs.* SIVF	0.001	0.006	0.049	0.00–0.38	↓	Suppl.7
	CG/telBB	RIF *vs.* SIVF	0.003	0.023	14.540	2.00–191.36	↑	Suppl.7
ERAP1 rs26653 G>C/HLA-C/KIR	GG/C2C2/AA+	ALL *vs.* Fertile	0.003	0.025	0.093	0.01–0.54	↓	Suppl.13
	GG/C2C2/AA+	RIF *vs.* Fertile	0.004	0.036	0.084	0.01–0.55	↓	Suppl.13
	CG/C2C2/AA+	ALL *vs.* Fertile	0.002	0.020	17.289	2.08–823.60	↑	Suppl.13
	CG/C2C2/AA+	RIF *vs.* Fertile	0.001	0.008	25.017	2.69–1275.24	↑	Suppl.13
ERAP1 rs26618 T>C/HLA-C	TT/C2+	RIF *vs.* Fertile	0.005	0.029	1.741	1.16–2.61	↑	Suppl.5
ERAP1 rs26618 T>C/KIR	TT/cenBB	RIF *vs.* Fertile	0.005	0.045	0.239	0.07–0.72	↓	Suppl.6
ERAP1 rs26618 T>C/HLA-C/KIR	CC/C2+/AA+	ALL *vs.* Fertile	0.005	0.031	0.084	0.00–0.66	↓	Suppl.13
ERAP1 rs2287987 T>C/KIR	CT/telBB	ALL *vs.* Fertile	0.001	0.007	0.111	0.02–0.46	↓	Suppl.10
	CT/telBB	RIF *vs.* Fertile	0.002	0.016	0.055	0.00–0.47	↓	Suppl.10
ERAP1 rs6861666 A>G/KIR	AA/cenBB	ALL *vs.* Fertile	0.001	0.010	7.435	1.87–43.47	↑	Suppl.11
	AA/cenBB	RIF *vs.* Fertile	0.003	0.029	12.373	1.68–553.07	↑	Suppl.11
	AG/cenBB	ALL *vs.* Fertile	0.002	0.020	0.149	0.03–0.60	↓	Suppl.11
ERAP2 rs2248374 A>G/KIR	GG/cenAB/telAB	RIF *vs.* SIVF	0.002	0.051	6.262	1.66–35.58	↑	Suppl.12

↑, susceptibility; ↓, protection; IVF-ET, in vitro fertilization embryo transfer; RIF, recurrent implantation failure; SIVF, successful pregnancy after IVF-ET; ALL, all IVF patients; p, probability; p_corr.,_ probability after Bonferroni correction; OR, odds ratio; 95% CI, confidence interval from two-sided Fisher’s exact test.

First, we considered the differences in the *KIR*/*HLA-C* combinations between the studied groups. When we divided the patients with the AA genotype in terms of *HLA-C* genotypes, we observed that women positive for *KIR* genes in the Tel AA region and *HLA-C2C2* were more prevalent in IVF and RIF groups than in fertile women (p/p_corr._ = 0.009/0.026, OR = 2.020; p/p_corr._ = 0.004/0.012, OR = 2.321, respectively; **Supplementary Table 4**). Therefore, having a female *HLA-C2* allotype positive also for *KIR* genes in the Tel AA region is not favorable for becoming pregnant.

In subsequent stages of the analyses, we considered the differences in the frequency of individual *ERAP* in double and triple combinations with *KIR* and *HLA-C* genes. We have obtained many significant but weak statistical results. Analyses for *ERAP1* rs26653, rs26618, rs2287987, and rs6861666 deserve attention. Women carrying rs26653 ERAP1 GG/HLA-C2C2 combination are protected from infertility and RIF (p/p_corr._ = 0.002/0.022, OR = 0.343, and p/p_corr._ = 0.001/0.005, OR = 0.252, respectively; **Supplementary Table 5**), while those with CG/HLA-C2C2 are predisposed to RIF (p/p_corr._ = 0.002/0.016, OR = 3.661). The protection against infertility and RIF is also observed in women with GG ERAP1 rs26653/HLA-C2C2/KIR AA combination (p/p_corr._ = 0.003/0.025, OR = 0.093, and p/p_corr._ = 0.004/0.036, OR = 0.084, respectively; **Supplementary Table 13**).

Women positive for rs26618 TT and HLA-C2+ were more often observed in the RIF group than in the fertile group (p/p_corr._ = 0.005/0.029, OR = 1.741; **Supplementary Table 5**). On the other hand, carriers of the rs26618 CC and HLA-C2+ combination were protected against infertility despite the fact that they had the *KIR* AA+ genotype (p/p_corr._ = 0.005/0.031, OR = 0.084; **Supplementary Table 13**). However, when we considered only the rs26618 *ERAP1* and *KIR* combination, we found statistically significant differences between women with rs26618 TT and *KIR* genotypes from the Cen BB region. This combination was more common in the fertile control group than in the RIF group (p/p_corr._ = 0.005/0.045, OR = 0.239; **Supplementary Table 6**).

The rs2287987 ERAP1 CT/KIR Tel BB combination protects against infertility and RIF (p/p_corr._ = 0.001/0.007, OR = 0.111, and p/p_corr._ = 0.002/0.016, OR = 0.055, respectively; **Supplementary Table 10**).

Carriers of *ERAP1* rs6861666 AA and *KIR* in the BB centromeric region are at risk of infertility and RIF (p/p_corr._ = 0.001/0.010, OR = 7.435, and p/p_corr._ = 0.003/0.029, OR = 12.373, respectively; **Supplementary Table 11**). In contrast, those with rs6861666 AG are less likely to experience infertility (p/p_corr._ = 0.002/0.020, OR = 0.149; **Supplementary Table 11**).

In the case of *ERAP2* rs2248374 and *KIR*, we found differences between the RIF and successful pregnancy after IVF-ETs (SIVF) groups in carriers of the GG/Cen AB/Tel AB combination (p/p_corr._ = 0.002/0.051, OR = 6.262; **Supplementary Table 12**). We did not find statistically significant results regarding comparisons of *ERAP* haplotype combinations with both HLA-C and KIR (**Supplementary Tables 14** and **15**).

Summarizing the genetic portion of this work, among the *ERAP* polymorphisms studied, rs26653 and rs26618 had the greatest influence on infertility susceptibility and RIF in HLA-C2 allotype-positive patients.

### Comparison of ERAP1 and ERAP2 Secretion in *In Vitro* Fertilization Patients and the Fertile Control Group

**Figure 1:**
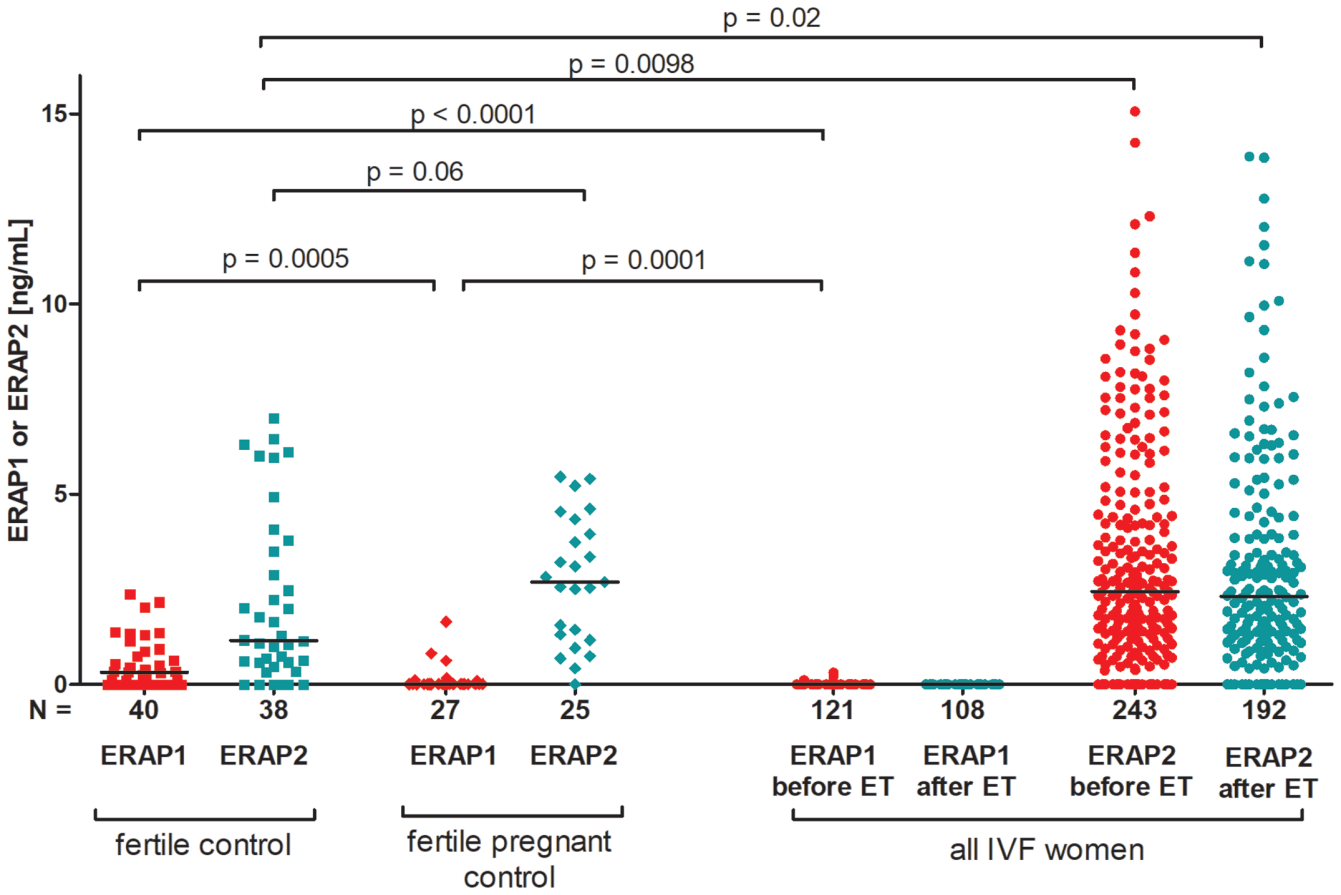
Comparison of ERAP1 and ERAP2 secretion in IVF patients and fertile control. Red points mean measurement of ERAP1; blue, ERAP2. IVF, *in vitro* fertilization; ET, embryo transfer.

We observed that fertile women who had given birth in the past secrete significantly more ERAP1 than women who underwent IVF, and women who were pregnant at the time of blood collection for the tests (p < 0.0001, median 0.316 *vs.* 0.000 ng/ml, respectively; and p = 0.0005, median 0.316 *vs.* 0.000 ng/ml, respectively; **Figure 1**). On the other hand, in the case of ERAP2, we observed the opposite situation; i.e., fertile women who had given birth in the past had statistically far less ERAP2 than IVF patients, regardless of whether the patient was before or after the ET (p = 0.0098, median 2.444 *vs.* 1.150 ng/ml, respectively; p = 0.02, median 2.444 *vs.* 2.311 ng/ml; **Figure 1**). ERAP2 levels were also increased in women who became pregnant naturally and were currently pregnant at the time of the study as compared with fertile women who had given birth in the past (p = 0.06, median 2.690 *vs.* 1.150 ng/ml, respectively; **Figure 1**).

### ERAP1 Secretion Impact on the Outcome of Pregnancy

**Figure 2:**
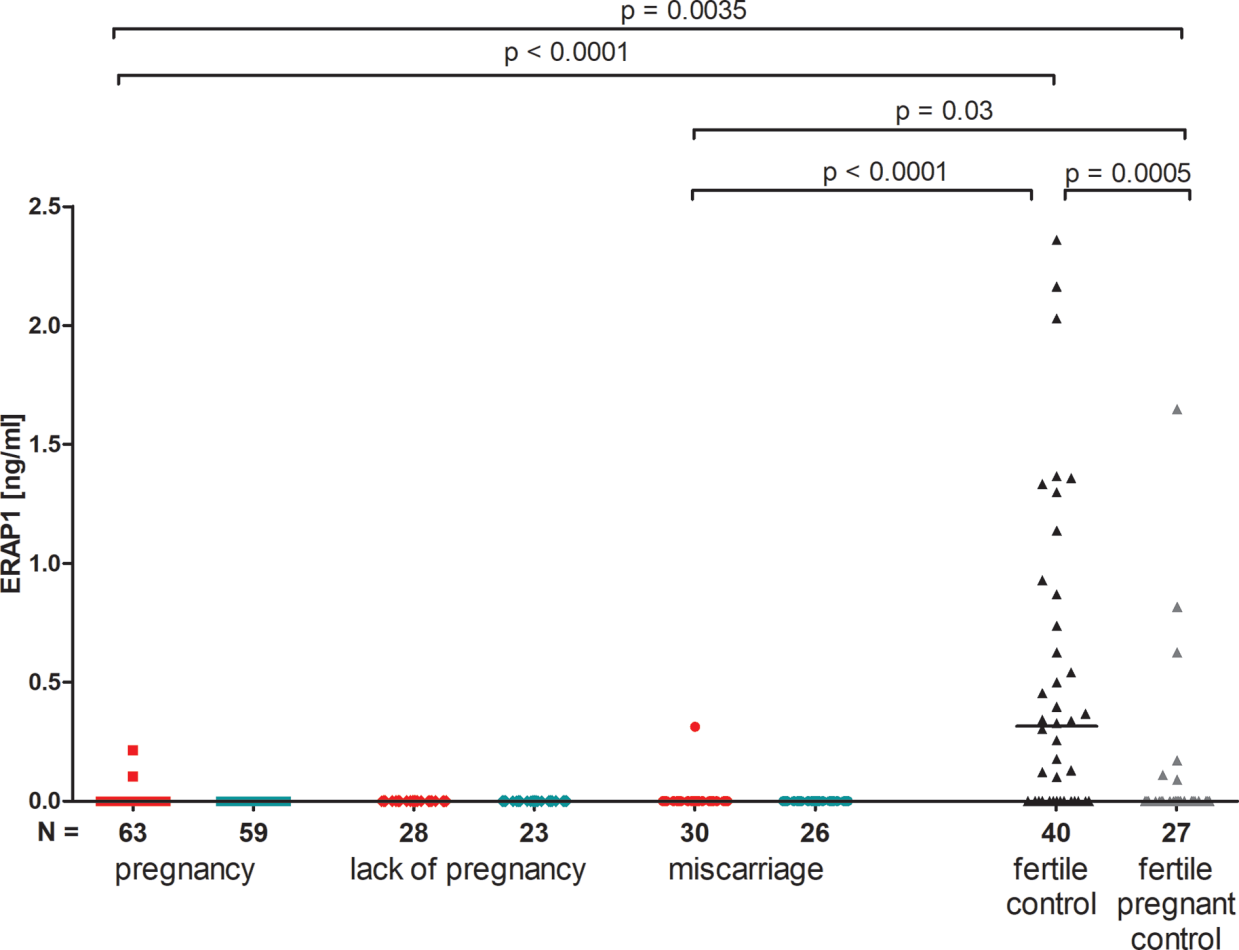
Impact of ERAP1 secretion on the pregnancy outcome. Red points mean measurement before embryo transfer; blue, after embryo transfer; black points, fertile control; gray points, fertile pregnant control.

IVF patients did virtually not secrete ERAP1; therefore, we found statistically significant differences between those patients and women from control groups. The following results were found: in patients with pregnancy after IVF *versus* fertile control group who had given birth in the past (p < 0.0001, median 0.000 *vs.* 0.316 ng/ml), and *versus* those women in a current natural pregnancy (p = 0.0035, median 0.000 *vs.* 0.000 ng/ml). Also patients who suffered a miscarriage after IVF-ET differed from control groups (p < 0.0001, median 0.000 *vs.* 0.316 ng/ml for women who had given birth in the past and p = 0.03, median 0.000 *vs.* 0.000 ng/ml for women with a current natural pregnancy) (**Figure 2**).

### ERAP2 Secretion Impact on the Pregnancy Outcome

**Figure 3:**
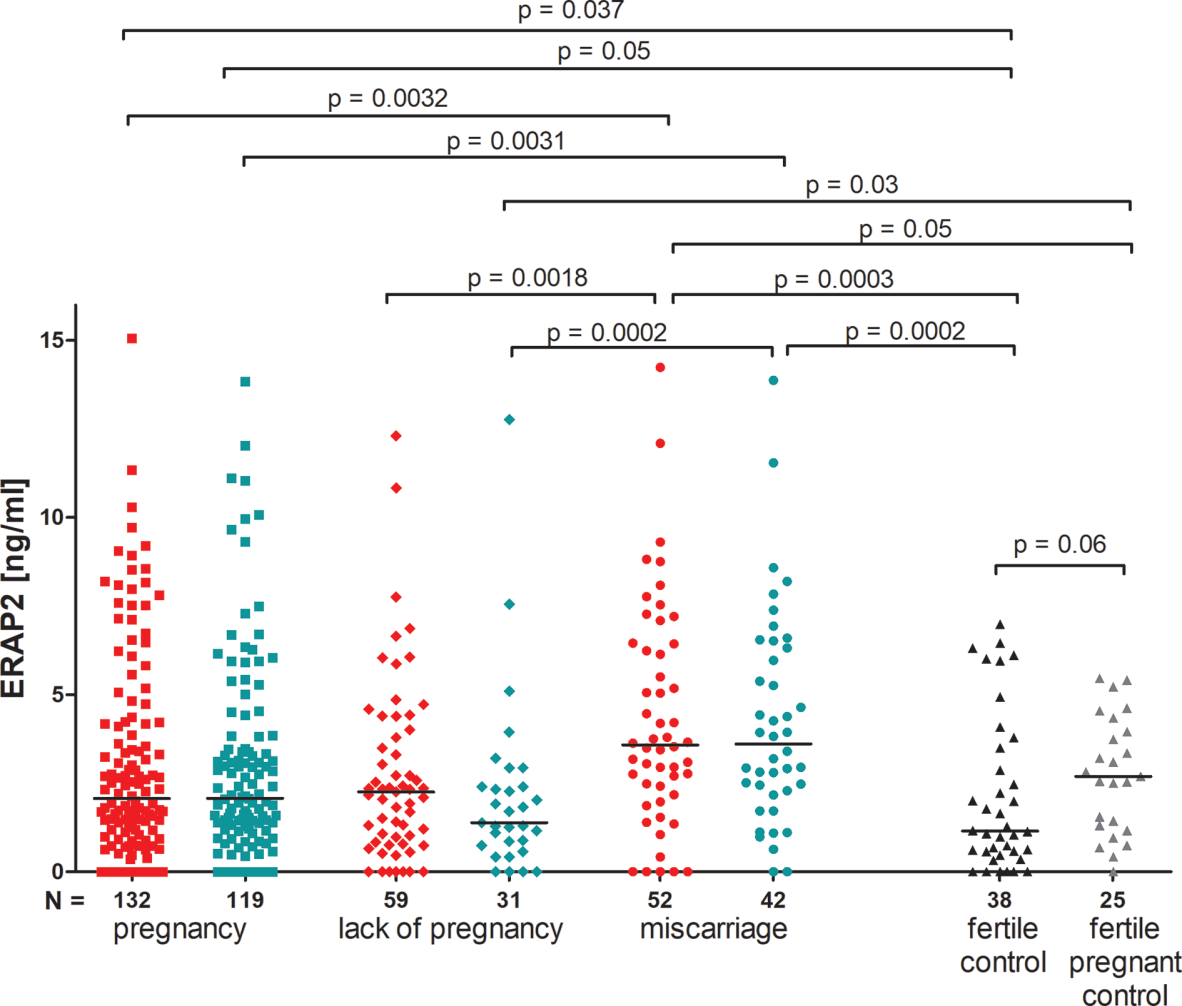
Impact of ERAP2 secretion on the pregnancy outcome. Red points mean measurement before embryo transfer; while blue, after embryo transfer; black points, fertile control; gray points, fertile pregnant.

The concentration of ERAP2 measured before and after ET did not differ in patients who became pregnant, as well as in those who miscarried. We observed a decrease in concentration after ET in patients who did not become pregnant, although not statistically significant (median 2.260 *vs.* 1.390 ng/ml; **Figure 3**). Moreover, patients who became pregnant after IVF-ET secrete far less ERAP2 than patients who miscarried (p = 0.0032, median 2.072 *vs.* 3.580 ng/ml; **Figure 3**). Both those patients who became pregnant after IVF-ET and those who miscarried were different from women having given birth in the past (p = 0.037, median 2.072 *vs.* 1.150 ng/ml and p = 0.0003, median 3.580 *vs.* 1.150 ng/ml, respectively; **Figure 3**). It should be emphasized that women who were pregnant during the ERAP2 level test had a median level of 2.690 ng/ml (**Figure 3**).

**Figure 4:**
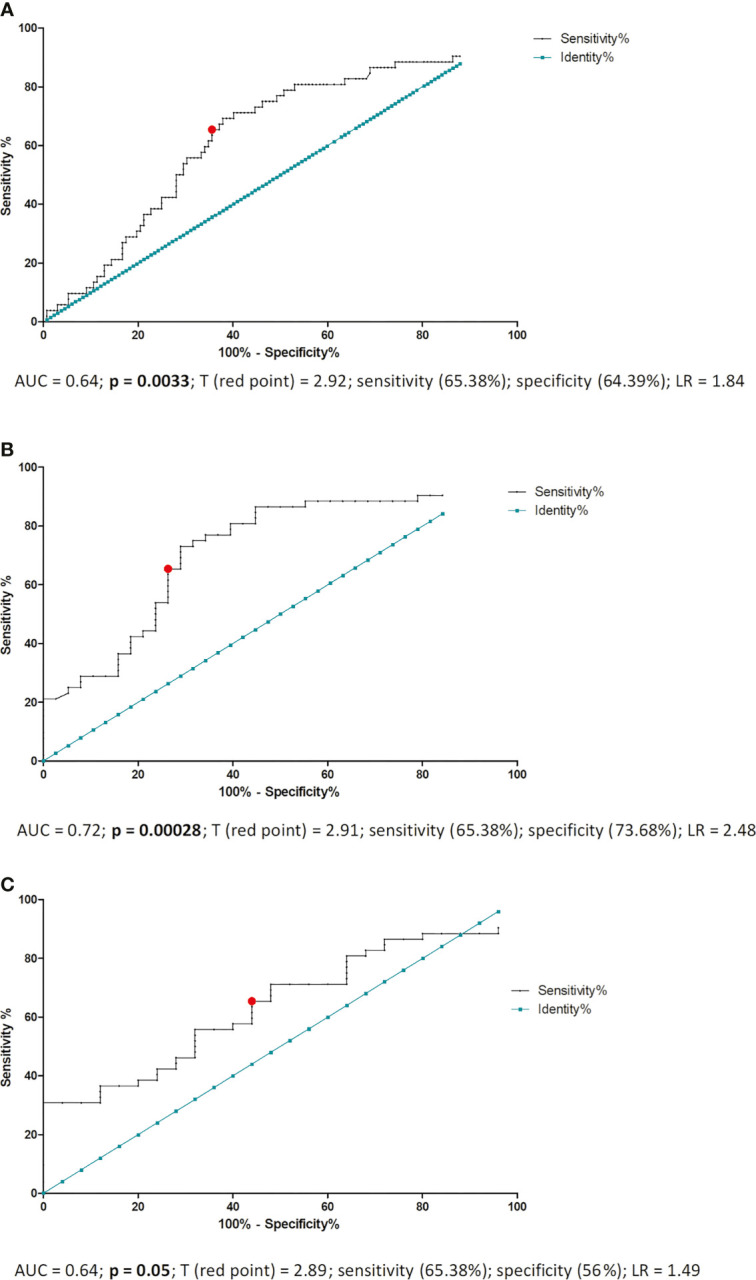
ROC analyses of ERAP2 secretion in IVF patients and fertile control. AUC, area under curve; p, probability value; T, threshold value; LR, likelihood ratio; IVF, *in vitro* fertilization; ROC, receiver operating characteristic. **(A)** ROC analysis of ERAP2 secretion into the plasma of pregnant patients and patients who miscarried after IVF-ET. IVF-ET, *in vitro* fertilization embryo transfer. **(B)** ROC analysis of ERAP2 secretion into the plasma of women who became pregnant naturally and gave birth to healthy child in the past and patients who miscarried after IVF-ET. **(C)** ROC analysis of ERAP2 secretion into the plasma of women who were pregnant from natural conception at the time of the test and patients who miscarried after IVF-ET.

ROC analysis was performed to determine the borderline ERAP2 value differentiating IVF patients with a successful pregnancy and patients who miscarried after IVF-ET. As a result of this analysis, we established 2.920 ng/ml of ERAP2 as the threshold value (area under the curve (AUC) = 0.64, p = 0.0033, sensitivity 65.38%, specificity 64.39%, and likelihood ratio (LR) = 1.84; **Figure 4A**). When we compared all fertile women (regardless of whether they had given birth in the past or are currently pregnant from natural fertilization) with those who miscarried after IVF-ET, the result of the ROC analysis turned out to be stronger (AUC = 0.72, p = 0.00028, sensitivity 65.38%, specificity 73.68%, and LR = 2.48), but the threshold value was similar to the previous analysis (2.910 ng/ml; **Figure 4B**). The third ROC analysis concerned the ERAP2 secretion of pregnant women from natural fertilization and patients who miscarried after IVF-ET. Threshold value was 2.890 ng/ml, AUC = 0.64, p = 0.05, sensitivity 65.38%, specificity 56%, and LR = 1.49; **Figure 4C**. All ROC analyses indicate a value of about 2.900 ng/ml as a point of differentiation between patients who miscarried and those who became pregnant and gave birth to healthy children.

## Discussion

We found the association between a female’s *KIR* genes from the telomeric region AA and *HLA-C2C2* with infertility as well as RIF in our population. In our study, there are twice as many patients with *KIRs* from Tel AA genotype (*KIR2DL4*, *KIR3DL1*, *KIR2DS4*, *KIR3DL2*) than in the control group (**Table 4** and **Supplementary Table 4**). Of these genes, two are framework genes (*KIR2DL4* and *KIR3DL2*). KIR3DL1 has no function in the development of pregnancy due to the lack of HLA-B ligand expression on trophoblast cells (47). On the other hand, *KIR2DS4* occurs in 80% of the Polish population in the form of a 22-nucleotide deletion that encodes a soluble protein, not a membrane receptor (44). Thus, the lack of activating genes, i.e., those found in haplotype B (including KIR2DS1), may be the reason for the lack of NK cell activation in Tel AA patients that would lead not to NK cytotoxicity but to the secretion of cytokines and growth factors that promote the development of pregnancy. Unfortunately, the analyses concerning Cen AA/Tel AA (AA genotype) lost their significance after the Bonferroni correction, but the percentage of patients in the RIF group and control women is also twice as high. Perhaps if the test groups were even greater in number, this significance would be maintained. Our study is consistent with the research by Hiby et al. (7), which showed a lower frequency of pregnancy-related diseases in the presence of the gene from the telomeric part of the KIR B haplotype — KIR2DS1. We were unable to demonstrate such an association with recurrent miscarriage after natural fertilization in a previously published study, precisely because of the even smaller sample size (45).

A higher miscarriage rate per cycle with own double ETs (DETs) in mothers with the *KIR* AA genotype compared with those with *KIR* AB and *KIR* BB genotypes was found by Alecsandru et al. (48). Also, a significantly decreased live birth rate per cycle was observed after DET using donated oocytes. Moreover, elective single ET (SET) was proposed to improve reproductive outcomes compared with DET (49). In our study, *KIR* AA genotype was significant only in *HLA-C2*-positive RIF patients, but again after Bonferroni correction, the significance was lost. It would seem that a group of almost 500 women is large, but in the case of such *KIR* gene polymorphisms and the combination with HLA-C, it is still insufficient. Summarizing, having *KIR* genes from haplotype A by the mother is unfavorable for embryo implantation, because receptors from this haplotype, when bound to the appropriate HLA-C2 ligand on trophoblast cells, inhibit NK cells. As a result, cytokines and growth factors promoting pregnancy development are not secreted. The risk of pregnancy disease is the greatest if the fetal HLA-C2 is inherited from the father (7, 50).

In general, there is little research into the association of *ERAP* polymorphism and reproductive diseases. The only pregnancy-related disease in which the role of ERAP has been studied is PE. Johnson et al. presented a weak association for *ERAP1*, and a significant association for *ERAP2* with PE susceptibility in Norwegian and Australian cohorts (51). In the Australian cohort, an association for the *ERAP1* gene (rs3734016) and for the *ERAP2* gene (rs2549782) was found. In turn, in the Norwegian cohort, associations for other *ERAP1* rs34750 and *ERAP2* rs17408150 polymorphisms were observed. However, they did not detect any associations with PE for rs27044, rs26653, and rs26618 of *ERAP1* and rs2248374 of *ERAP2* as we did for RIF and infertility. rs30187 of *ERAP1* seems to play a role in susceptibility to recurrent miscarriage after natural fertilization. This effect was strengthened in women with the genotype *KIR* Bx and *HLA-C2* and was shown in our own research (45). However, we have not demonstrated the role of this polymorphism in RIF after artificial fertilization.

In addition, a case–control study in Chilean and African American samples was reported by Hill et al. for associations between two SNPs in *ERAP2*, rs2549782, and rs17408150 with PE (52). An increased risk for PE in the African American population, but not in the Chilean population, was found. In addition, fetal *ERAP2* SNP rs2549782 polymorphism was associated with a higher risk for PE in African American women (53).

Some of the combinations of the *ERAP*, *KIR*, and *HLA-C* genes and their association with infertility and RIF need to be discussed, as there is no published research to date on the effect of such complex variants on reproductive success. Women carrying the rs26653 ERAP1 GG/HLA-C2C2 combination were protected from infertility and RIF, while those with GC/HLA-C2C2 were predisposed. The protective effect deepened when the woman in terms of *KIR* genotype was AA (the most inhibiting NK function) (**Table 4**, **Supplementary Tables 5** and **13**). Perhaps the GG rs26653 *ERAP1* genotype in *HLA-C2C2*- and *KIR* AA-positive women had an impact on the production of peptides that were not of optimal length (8–10 residues), meaning there was no proper interaction between self-HLA-C and KIRs from haplotype A. The role of maternal, self-HLA-C in regulation of dNK responsiveness was presented in a study by Sharkey et al. (54). They detected the expression and function of five inhibitory NK receptors in dNK, which were influenced by maternal HLA-C. Moreover, they found a decreased expression frequency of the cognate receptor, KIR2DL1, in dNK cells isolated from women carrying a HLA-C2 epitope. Production of the defective peptides by individuals with *ERAP1* rs26653 GG in *HLA-C2C2* and *KIR* AA women may lead to a lack of NK cell inhibition by KIR2DL1. However, we do not consider a possible semi-allogeneic response here because we did not analyze the partner’s HLA-C in this research. We will be able to draw such a conclusion when the analyses include the partner’s HLA-C and, additionally, their *ERAP* polymorphism.

Women positive for *ERAP1* rs26618 TT and *HLA-C2* were more often observed in the RIF group than in the fertile group, while the opposite was observed with CT and *HLA-C2* (**Table 4** and **Supplementary Table 5**). However, we observed that the TT genotype of *ERAP1* rs26618 in combination with *KIR* Cen BB was more frequently observed in the fertile control group than in IVF patients (**Table 4** and **Supplementary Table 6**). Cen-BB genotype means the presence of *KIR2DS2* and *KIR2DL2* and maybe *KIR2DL5B*, *KIR2DS3*, and *KIR2DL1*, suggesting their impact on protection against RIF when a woman is positive for the rs26618 TT genotype.

Moreover, carriers of the rs26618 CC and *HLA-C2* combination were protected against infertility despite the fact that they had the *KIR* AA genotype (**Table 4** and **Supplementary Table 13**). The *ERAP1* rs26618 affects the efficiency of a precursor peptide trimming for the HLAC*05-bound epitope, and it was displayed in a fraction of atopic dermatitis patients (36). Moreover, rs26618C (276Met) is a component of ERAP1 haplotypes of low enzymatic activity (55, 56). Low activity can affect the processing efficiency of the antigen and result in the production of an altered repertoire of peptides that cannot fit the HLA-C2 molecules. This can lead to a lack of inhibition during the HLA-C2 and KIR AA interactions, which is desirable for the development of pregnancy. If rs26618 TT provides the proper enzymatic activity, we can expect that the resulting antigenic peptides should guarantee the interaction with HLA-C2 and KIR AA and thus predispose to RIF, but it should protect in the combination with KIRs from Cen BB genotype.

Carriers of the genotype *ERAP1* rs6861666 AA and *KIR* Cen BB are predisposed to infertility and RIF. On the other hand, women with the rs6861666 AG genotype in combination with KIR Cen BB are protected against infertility (**Table 4** and **Supplementary Table 11**). rs6861666 indirectly affects the expression of both ERAP1 and ERAP2 (37).

In turn, the rs2287987 ERAP1 CT/KIR Tel BB combination protects against infertility and RIF (**Table 4** and **Supplementary Table 10**). The polymorphic amino acid of ERAP1 is located near the catalytic site (residue M349V) and therefore could influence the interaction of the enzyme on the substrate (35). In this case, we can only suppose that this combination, on the one hand, has *KIR* genes from the Tel BB region (favorable for pregnancy) and, on the other hand, the CT ERAP1 genotype, which will be responsible for correct substrate interaction (precursor peptide) with the enzyme.

It is also worth noting that in the case of ERAP2 rs2248374 and KIR combination analysis, we found differences between the RIF and SIVF groups in carriers of the rs2248374 GG/Cen AB/Tel AB combination. Patients who gave birth after IVF-ET have statistically less of such a combination than patients with RIF, while for those with rs2248374 AA, the opposite was observed (**Table 4** and **Supplementary Table 12**). In general, the *ERAP2* gene appears in two haplotypes: A and B. Haplotype A contains the rs2248374-A allele, while haplotype B contains the rs2248374-G allele. The ERAP2 mRNA derived from haplotype A encodes a full-length ERAP2 protein. In contrast, ERAP2 mRNA derived from haplotype B produces a truncated protein lacking catalytic domain and therefore without function (38). In the above-mentioned combination, patients are predisposed to RIF despite having a favorable *KIR* gene set (Cen AB/Tel AB) for the development of pregnancy; perhaps their *ERAP2* gene encodes an inactive form of the enzyme.

Summarizing this part of the Discussion, ERAP aminopeptidases cut peptides to the appropriate length, which are then presented in the context of HLA-C to KIR receptors; i.e., they have a direct role in shaping the correct HLA-C molecule, later indirectly influencing the interaction with KIR. Therefore, we believe that the *ERAP* genotype combination with HLA-C is more important than the combination with KIR. We believe that among the *ERAP* polymorphisms studied, rs26653 and rs26618 have the greatest influence on infertility susceptibility and RIF in HLA-C2-positive patients.

It is extremely interesting that the aminopeptidases that reside in the ER are secreted into peripheral blood. We discovered, for the first time, differences in the secretion of ERAP1 and ERAP2 into the plasma of patients who underwent an IVF procedure and fertile women who became pregnant naturally. Moreover, elevated levels of ERAP2, above 2.9 ng/ml, indicated a miscarriage.

There are reports of a multifunctional role for ERAP1 and ERAP2. ERAPs are involved in a variety of biological processes including the final trimming of peptides in the ER for presentation on MHC class I molecules (57), shedding of several cytokine receptors (58, 59), postnatal angiogenesis, and regulation of blood pressure (60). In addition, there are reports showing that they can trim receptors for proinflammatory cytokines such as TNF-α and the type I IL-6 cytokine receptor (IL-6Rα) and the type II IL-1 decoy receptor (IL-1RII) (59). Cui et al. also reported that ERAP1 binds to the extracellular domain of the TNFR1, facilitating TNFR1 shedding through the formation of a TNFR1/ERAP1 complex (58). The authors showed that overexpression of ERAP1 produces a soluble TNFR1 that competes with TNF receptors on the cell surface, thereby weakening the bioactivity of TNF-α when ERAP1 levels are elevated and restoring TNF-α when levels decline. TNF receptor shedding may also decrease the number of cell-surface receptors available for ligand binding. Thus, overexpression of ERAP1 would attenuate inflammation. Surprisingly, our IVF patients did not secrete ERAP1. The lack of ERAP1 secretion in IVF patients can be explained by the fact that the patients were taking steroids during the IVF procedure. Steroids have an anti-inflammatory effect. We can assume that their use reduces TNF-α (30) and IFN-γ levels (29), which are necessary for the expression and activity of ERAP1. Indeed, 85% and 95% of our patients who were tested for ERAP1 in plasma did not secrete IFN-γ and TNF-α, respectively. For patients who were tested for the level of ERAP2, the percentage was 76% and 95%, respectively (our unpublished data). Two questions arise: Does it make sense to use steroids in all IVF patients? The adjuvant administration of steroids in women undergoing controlled IVF/intracytoplasmic sperm injection (ICSI) cycles is unclear (61, 62). Only patients with the higher levels of IFN-γ and TNF-α should be considered to benefit from glucocorticoid adjuvant therapy. However, further clinical trials are needed.

And the second question is: Why did steroids suppress the secretion of ERAP1 and not ERAP2? Studies by Saveanu et al. (63) and Evnouchidou et al. (64) reported a physical interaction between ERAP1 and ERAP2, which results in a shift of their enzymatic properties and an increased efficiency in processing antigenic precursors (63, 64). In addition, a study by Tanioka et al. showed that ERAP2 (L-RAP) might compensate for the knockdown of ERAP1 in trimming antigenic peptides. ERAP2 was the dominant trimming enzyme in tissues with low ERAP1 expression, which mimics the effect of ERAP1 siRNA treatment (65). Perhaps in our IVF patients, a similar situation exists. In the case of silencing the expression and secretion of ERAP1, it is ERAP2 that takes over. However, overexpression of ERAP2 (as in our patients with miscarriage) is unfavorable.

Studies in syngeneic mice indicate rejection of T-cell lymphoma RMA following the inhibition of ERAP1 through a tumor-specific NK cell response due to impaired pMHC class I engagement of Ly49C/I NK cell-inhibitory receptors (66). Ly49 receptors are the equivalent of KIR receptors in humans. Moreover, elimination of ERAP1 from LIF-treated human choriocarcinoma cell line, JEG-3 cells, reduced the cell surface HLA-G1 expression and soluble HLA-G1 secretion. ERAP1 was localized in the ER of trophoblasts and involved in the regulation of cell surface HLA-G expression (67). Therefore, ERAP1 may have an effect on the peptide repertoire presented in the context of HLA-G1 in trophoblasts. Our research on sHLA-G secretion in IVF patients may prove this thesis. Patients who did not become pregnant or who experienced a miscarriage secreted significantly less sHLA-G than patients who gave birth as a result of IVF-ET (41), although it should be noted that Xu et al. showed that ERAP1 expression was significantly elevated in placental tissues of PE and that hypoxia increased ERAP1 expression in trophoblasts (68). However, in our study, ERAP1 secretion seems desirable because we have demonstrated its presence in the plasma of fertile women who gave birth in the past. A lower ERAP1 secretion, but nevertheless secretion, was observed in pregnant women after natural conception. Aldhamen et al. (69) analyzed the functional properties of secreted ERAP1. They confirmed secretion of ERAP1 from RAW264.7 cells upon lipopolysaccharide (LPS)-induced IFN-γ. Moreover, they treated peripheral blood mononuclear cells with catalytically active ERAP1 and showed activation of NK cells, dendritic cells, and T cells (69). This shows that when ERAP1 is secreted outside the cell, it has an effect on the activation of immune cells, including NK cells.

Our study on the secretion of ERAP2 into plasma suggests that the ERAP2 protein is needed for proper embryo implantation, especially when ERAP1 expression is suppressed. This is confirmed by the fact that women pregnant from natural conception secrete ERAP2. Since the secretion of ERAP2 is necessary for the proper development of a pregnancy, one final question arises: What percentage of ERAP2 released into the blood of patients and fertile controls is enzymatically active? We think this will be the topic of our next research as soon as we collect new plasma samples.

Thus, the expression and secretion of both ERAP1 and ERAP2 must be optimal for the correct implantation and development of the embryo. Our genetic and protein study on ERAPs indicates that both ERAP1 and ERAP2 may be involved in processes related to reproduction, including RIF.

## Data Availability Statement

The data analyzed in this study is subject to the following licenses/restrictions: data collections can be made available upon request. For now, however, we do not want to make them public due to the planned patent solutions. Requests to access these datasets should be directed to IN, (izabela.nowak@hirszfeld.pl).

## Ethics Statement

The studies involving human participants were reviewed and approved by Local Ethics Committee of Mothers’ Memorial Hospital—Research Institute in Łódź. The patients/participants provided their written informed consent to participate in this study.

## Author Contributions

All authors were involved in the conception and design of the study, data interpretation and analysis, and manuscript generation. KP, AT, AW, and IN were involved in data acquisition, generating the bulk of the data, performing the data analysis, carrying out the statistical analysis, and drafting the article. PR, MR, JW, and AM were essential for all aspects of obtaining the human samples and were involved in manuscript generation. All authors contributed to the article and approved the submitted version.

## Funding

Part of the research on genotyping of KIR and HLA-C was financed by the grant of the National Science Centre No. 2014/13/B/NZ5/00273, while the research on ERAP was covered by grant No. 2018/29/N/NZ5/00940.

## Conflict of Interest

The authors declare that the research was conducted in the absence of any commercial or financial relationships that could be construed as a potential conflict of interest.

## Publisher’s Note

All claims expressed in this article are solely those of the authors and do not necessarily represent those of their affiliated organizations, or those of the publisher, the editors and the reviewers. Any product that may be evaluated in this article, or claim that may be made by its manufacturer, is not guaranteed or endorsed by the publisher.
