# Supplementary material for: ERAP, KIR, and HLA-C Profile in Recurrent Implantation Failure
Source: Front Immunol. 2021 Oct 22;12:755624. doi: 10.3389/fimmu.2021.755624 (PMC8569704; doi:10.3389/fimmu.2021.755624)
Supplement: Supplementary file 1 [file Table_1.docx]

**Supplementary Table 1.** Individual KIR, KIR genotypes and HLA-C frequencies in fertile women and women who participated in IVF.

| **KIR /KIR genotype/**  **HLA-C genotype** | **All IVF** | | **RIF** | | **SIVF** | | **Fertile** | |
| --- | --- | --- | --- | --- | --- | --- | --- | --- |
|  | N |  | N |  | N |  | N |  |
| 2DL1 | 495 | 472 (95.35) | 283 | 273 (96.47) | 161 | 153 (95.03) | 385 | 365 (94.81) |
| 2DL2 | 493 | 291 (59.03) | 282 | 168 (59.57) | 160 | 96 (60.00) | 384 | 203 (52.86) |
| 2DL3 | 496 | 441 (88.91) | 283 | 253 (89.40) | 161 | 143 (88.82) | 385 | 339 (88.05) |
| 2DL4 norm. | 493 | 332 (67.34) | 281 | 197 (70.11) | 161 | 102 (63.35) | 385 | 264 (68.57) |
| 2DL4 del. | 491 | 377 (76.78) | 280 | 220 (78.57) | 160 | 116 (72.50) | 385 | 299 (77.66) |
| 2DL5 all | 496 | 264 (53.23) | 283 | 151 (53.36) | 161 | 83 (51.55) | 385 | 207 (53.77) |
| 2DL5 gr.1 | 493 | 140 (28.40) | 279 | 80 (28.67) | 161 | 47 (29.19) | 363 | 109 (30.03) |
| 2DL5 gr.2 | 493 | 169 (34.28) | 282 | 97 (34.40) | 161 | 51 (31.68) | 363 | 118 (32.51) |
| 2DS1 | 496 | 204 (41.13) | 283 | 114 (40.28) | 161 | 70 (43.48) | 385 | 169 (43.90) |
| 2DS2 | 496 | 297 (59.88) | 283 | 171 (60.42) | 161 | 98 (60.87) | 385 | 212 (55.06) |
| 2DS3 | 496 | 169 (34.07) | 283 | 96 (33.92) | 161 | 50 (31.06) | 385 | 134 (34.81) |
| 2DS4 norm. | 495 | 158 (31.92) | 283 | 94 (33.22) | 161 | 48 (29.81) | 385 | 112 (29.09) |
| 2DS4 del. | 495 | 408 (82.42) | 283 | 238 (84.10) | 160 | 128 (80.00) | 385 | 323 (83.90) |
| 2DS5 | 494 | 143 (28.95) | 282 | 81 (28.72) | 160 | 48 (30.00) | 385 | 123 (31.95) |
| 3DL1 | 494 | 463 (93.72) | 282 | 267 (94.68) | 161 | 148 (91.93) | 384 | 360 (93.75) |
| 3DL2 | 495 | 492 (99.39) | 283 | 282 (99.65) | 160 | 159 (99.38) | 383 | 383 (100.00) |
| 3DL3 | 496 | 496 (100.00) | 283 | 283 (100.00) | 161 | 161 (100.00) | 383 | 383 (100.00) |
| 3DS1 | 496 | 177 (35.69) | 283 | 103 (36.40) | 161 | 55 (34.16) | 384 | 153 (39.84) |
| 2DP1 | 488 | 474 (97.13) | 279 | 272 (97.49) | 160 | 156 (97.50) | 364 | 349 (95.88) |
| 3DP1 norm. | 489 | 469 (95.91) | 279 | 271 (97.13) | 161 | 154 (95.65) | 363 | 346 (95.32) |
| 3DP1 var. | 490 | 150 (30.61) | 279 | 90 (32.26) | 161 | 50 (31.06) | 363 | 106 (29.20) |
| AA | 496 | 138 (27.82) | 283 | 77 (27.21) | 161 | 44 (27.33) | 385 | 110 (28.57) |
| Bx | 496 | 358 (72.18) | 283 | 206 (72.79) | 161 | 117 (72.67) | 385 | 275 (71.43) |
| cenAA | 496 | 197 (39.72) | 283 | 112 (39.58) | 161 | 61 (37.89) | 385 | 173 (44.94) |
| cenAB | 496 | 244 (49.19) | 283 | 141 (49.82) | 161 | 82 (50.93) | 385 | 166 (43.12) |
| cenBB | 496 | 55 (11.09) | 283 | 30 (10.60) | 161 | 18 (11.18) | 385 | 46 (11.95) |
| telAA | 496 | 286 (57.66) | 283 | 164 (57.95) | 161 | 90 (55.90) | 384 | 205 (53.39) |
| telAB | 496 | 177 (35.69) | 283 | 103 (36.40) | 161 | 58 (36.02) | 384 | 156 (40.63) |
| telBB | 496 | 33 (6.65) | 283 | 16 (5.65) | 161 | 13 (8.07) | 384 | 23 (5.99) |
| cenAA/telAA | 197 | 137 (69.54) | 112 | 77 (68.75) | 61 | 43 (70.49) | 172 | 110 (63.95) |
| cenAA/telAB | 197 | 55 (27.92) | 112 | 33 (29.46) | 61 | 15 (24.59) | 172 | 58 (33.72) |
| cenAA/telBB | 197 | 5 (2.54) | 112 | 2 (1.79) | 61 | 3 (4.92) | 172 | 4 (2.33) |
| cenAB/telAA | 244 | 125 (51.23) | 141 | 72 (51.06) | 82 | 42 (51.22) | 166 | 79 (47.59) |
| cenAB/telAB | 244 | 102 (41.80) | 141 | 60 (42.55) | 82 | 36 (43.90) | 166 | 76 (45.78) |
| cenAB/telBB | 244 | 17 (6.97) | 141 | 9 (6.38) | 82 | 4 (4.88) | 166 | 11 (6.63) |
| cenBB/telAA | 55 | 24 (43.64) | 30 | 15 (50.00) | 18 | 5 (27.78) | 46 | 16 (34.78) |
| cenBB/telAB | 55 | 20 (36.36) | 30 | 10 (33.33) | 18 | 7 (38.89) | 46 | 22 (47.83) |
| cenBB/telBB | 55 | 11 (20.00) | 30 | 5 (16.67) | 18 | 6 (33.33) | 46 | 8 (17.39) |
| C1 | 990 | 570 (57.58) | 566 | 328 (57.95) | 322 | 186 (57.76) | 766 | 457 (59.66) |
| C2 | 990 | 420 (42.42) | 566 | 238 (42.05) | 322 | 136 (42.24) | 766 | 309 (40.34) |
| C1C1 | 495 | 162 (32.73) | 283 | 96 (33.92) | 161 | 52 (32.30) | 383 | 135 (35.25) |
| C1C2 | 495 | 246 (49.70) | 283 | 136 (48.06) | 161 | 82 (50.93) | 383 | 187 (48.83) |
| C2C2 | 495 | 87 (17.58) | 283 | 51 (18.02) | 161 | 27 (16.77) | 383 | 61 (15.93) |

IVF-ET – in vitro fertilization embryo transfer; RIF – recurrent implantation failure; SIVF – successful pregnancy after IVF-ET. Values in parentheses are in percentages.
