# Supplementary material for: ERAP, KIR, and HLA-C Profile in Recurrent Implantation Failure
Source: Front Immunol. 2021 Oct 22;12:755624. doi: 10.3389/fimmu.2021.755624 (PMC8569704; doi:10.3389/fimmu.2021.755624)
Supplement: Supplementary file 2 [file Table_2.docx]

**Supplementary Table 2** ERAP1 and ERAP2 genotypes and minor allele frequencies in women from fertile control and patient groups.

|  | **All IVF** | **RIF** | **SIVF** | **Fertile** |
| --- | --- | --- | --- | --- |
| **ERAP1 rs30187** | N = 496 | N = 283 | N = 161 | N = 385 |
| CC | 221 (44.56) | 118 (41.70) | 75 (46.58) | 185 (48.05) |
| CT | 219 (44.15) | 130 (45.94) | 70 (43.48) | 166 (43.12) |
| TT | 56 (11.29) | 35 (12.36) | 16 (9.94) | 34 (8.83) |
| Minor allele T | 331 (33.37) | 200 (35.34) | 102 (31.68) | 234 (30.39) |
| H-W | p = 0.875 | p = 0.930 | p = 0.955 | p = 0.708 |
| **ERAP1 rs27044** | N = 496 | N = 283 | N = 161 | N = 385 |
| CC | 264 (53.23) | **141 (49.82**)**^a^** | 88 (54.66) | 225 (58.44) |
| CG | 197 (39.72) | **120 (42.40)^b^** | 63 (39.13) | 133 (34.55) |
| GG | 35 (7.05) | 22 (7.78) | 10 (6.21) | 27 (7.01) |
| Minor allele G | 267 (26.92) | 164 (28.98) | 83 (25.78) | 187 (24.29) |
| H-W | p = 0.832 | p = 0.229 | p = 0.774 | p = 0.234 |
| **ERAP1 rs26653** | N = 496 | N = 283 | N = 161 | N = 382 |
| GG | 270 (54.44) | 149 (52.65) | 89 (55.28) | 213 (55.76) |
| CG | 201 (40.52) | 120 (42.40) | 66 (40.99) | 150 (39.27) |
| CC | 25 (5.04) | 14 (4.95) | 6 (3.73) | 19 (4.97) |
| Minor allele C | 251 (25.30) | 148 (26.15) | 78 (24.22) | 188 (24.61) |
| H-W | p = 0.109 | p = 0.100 | p = 0.139 | p = 0.255 |
| **ERAP1 rs26618** | N = 496 | N = 283 | N = 161 | N = 385 |
| TT | 268 (54.03) | **163 (57.60)^c^** | 81 (50.31) | 190 (49.35) |
| CT | 188 (37.90) | 99 (34.98) | 65 (40.37) | 162 (42.08) |
| CC | 40 (8.07) | 21 (7.42) | 15 (9.32) | 33 (8.57) |
| Minor allele C | 268 (27.02) | 141 (24.91) | 95 (29.50) | 228 (29.61) |
| H-W | p = 0.387 | p = 0.275 | p = 0.709 | p = 0.853 |
| **ERAP1 rs2287987** | N = 496 | N = 283 | N = 161 | N = 385 |
| TT | 307 (61.90) | 175 (61.84) | 103 (63.98) | 228 (59.22) |
| CT | 165 (33.27) | 94 (33.22) | 50 (31.06) | 146 (37.92) |
| CC | 24 (4.83) | 14 (4.94) | 8 (4.96) | 11 (2.86) |
| Minor allele C | 213 (21.47) | 122 (21.55) | 66 (20.50) | 168 (21.82) |
| H-W | p = 0.763 | p = 0.765 | p = 0.550 | p = 0.029 |
| **ERAP2 rs2248374** | N = 495 | N = 282 | N = 161 | N = 382 |
| AA | 126 (25.45) | 67 (23.76) | 48 (29.81) | 106 (27.75) |
| AG | 237 (47.88) | 134 (47.52) | 76 (47.20) | 181 (47.38) |
| GG | 132 (26.67) | 81 (28.72) | 37 (22.99) | 95 (24.87) |
| Minor allele G | 501 (50.61) | 296 (52.48) | 150 (46.58) | 371 (48.56) |
| H-W | p = 0.347 | p = 0.459 | p = 0.514 | p = 0.313 |
| **ERAP1 rs6861666** | N = 495 | N = 282 | N = 161 | 380 |
| AA | 424 (85.66) | 238 (84.40) | 138 (85.71) | 326 (85.79) |
| AG | 69 (13.94) | 43 (15.25) | 22 (13.66) | 53 (13.95) |
| GG | 2 (0.40) | 1 (0.35) | 1 (0.63) | 1 (0.26) |
| Minor allele G | 73 (7.37) | 45 (7.98) | 24 (7.45) | 55 (7.24) |
| H-W | p = 0.649 | p = 0.519 | p = 0.904 | p = 0.449 |

IVF-ET – in vitro fertilization embryo transfer; RIF – recurrent implantation failure; SIVF – successful pregnancy after IVF-ET; H-W – Hardy-Weinberg equilibrium; p – probability; OR – odds ratio; 95% CI – confidence interval from two-sided Fisher’s exact test. Values in bold indicate significant differences. Values in parentheses are in percentages.

**RIF vs. Fertile:** ^a^p = 0.028, OR = 0.706, 95% CI (0.51-0.97); ^b^p = 0.044, OR = 1.394, 95% CI (1.00-1.94); ^c^p = 0.041, OR = 1.393, 95% CI (1.01-1.92)
