# Supplementary material for: ERAP, KIR, and HLA-C Profile in Recurrent Implantation Failure
Source: Front Immunol. 2021 Oct 22;12:755624. doi: 10.3389/fimmu.2021.755624 (PMC8569704; doi:10.3389/fimmu.2021.755624)
Supplement: Supplementary file 3 [file Table_3.docx]

**Supplementary Table 3** ERAP haplotype frequencies in women from fertile control and patient groups.

| **ERAP**  **haplotype** | **SNP order** | **All IVF** | **RIF** | **SIVF** | **Fertile** |
| --- | --- | --- | --- | --- | --- |
|  |  | N = 990 | N = 564 | N = 322 | N = 764 |
| H1 | A-A-G-C-T-C-C | 224 (22.63) | **115 (20.39)^a^** | 85 (26.41) | 184 (24.09) |
| H2 | A-A-G-T-C-C-C | 110 (11.11) | 57 (10.11) | 42 (13.04) | 91 (11.92) |
| H3 | G-A-C-T-T-T-G | 109 (11.01) | 65 (11.52) | 36 (11.18) | 78 (10.21) |
| H4 | G-A-G-T-C-C-C | 95 (9.60) | **61 (10.82)^b^** | 21 (6.52) | 73 (9.55) |
| H5 | G-A-G-T-T-C-C | 76 (7.68) | 43 (7.62) | 26 (8.07) | 62 (8.12) |
| H6 | G-A-G-T-T-T-G | 89 (8.99) | 51 (9.04) | 30 (9.32) | 59 (7.72) |
| H7 | G-A-G-C-T-C-C | 44 (4.44) | 26 (4.61) | 10 (3.11) | 40 (5.24) |
| H8 | A-A-C-T-T-T-C | 45 (4.55) | 26 (4.61) | 14 (4.35) | 39 (5.10) |
| H9 | A-A-G-T-T-C-C | 42 (4.24) | 24 (4.26) | 12 (3.73) | 24 (3.14) |
| H10 | A-A-C-T-T-C-C | 22 (2.22) | 13 (2.30) | 7 (2.17) | 24 (3.14) |
| H11 | G-G-C-T-T-C-C | 32 (3.23) | 20 (3.55) | 11 (3.42) | 21 (2.75) |
| H12 | G-G-G-T-T-T-G | 18 (1.82) | 12 (2.13) | 6 (1.86) | 16 (2.09) |
| H13 | A-G-G-T-T-T-G | 15 (1.52) | 11 (1.95) | 3 (0.93) | 11 (1.44) |
| H14 | A-A-G-T-T-T-G | 19 (1.92) | 13 (2.30) | 6 (1.86) | 10 (1.31) |
| H15 | G-A-C-T-T-C-C | 7 (0.71) | 3 (0.53) | 2 (0.62) | 9 (1.18) |
| H16 | G-A-C-T-T-T-C | **23 (2.32)^c^** | 12 (2.13) | 6 (1.86) | 8 (1.05) |
| H17 | A-A-C-T-T-T-G | 7 (0.71) | **7 (1.24)^d^** | 0 (0.00) | 4 (0.52) |
| H18 | G-G-C-T-T-T-G | 4 (0.40) | 1 (0.18) | 1 (0.31) | 4 (0.52) |
| H19 | G-A-G-T-C-C-G | 3 (0.30) | 1 (0.18) | 1 (0.31) | 1 (0.13) |
| H20 | A-A-G-T-C-C-G | 2 (0.20) | 2 (0.35) | 0 (0.00) | 1 (0.13) |
| H21 | A-G-C-T-T-T-C | 1 (0.10) | 1 (0.18) | 0 (0.00) | 1 (0.13) |
| H22 | A-A-G-T-T-T-C | 0 (0.00) | 0 (0.00) | 0 (0.00) | 1 (0.13) |
| H23 | A-G-G-C-T-C-G | 0 (0.00) | 0 (0.00) | 0 (0.00) | 1 (0.13) |
| H24 | A-G-G-T-C-C-G | 0 (0.00) | 0 (0.00) | 0 (0.00) | 1 (0.13) |
| H25 | A-G-G-T-C-T-C | 0 (0.00) | 0 (0.00) | 0 (0.00) | 1 (0.13) |
| H26 | A-G-G-T-C-C-C | 2 (0.20) | 0 (0.00) | 2 (0.62) | 0 (0.00) |
| H27 | A-G-C-T-T-C-C | 1 (0.10) | 0 (0.00) | 1 (0.31) | 0 (0.00) |

IVF-ET – in vitro fertilization embryo transfer; RIF – recurrent implantation failure; SIVF – successful pregnancy after IVF-ET; SNP – single nucleotide polymorphism; p – probability; p_corr._ – probability after Bonferroni correction for 27 possible haplotypes; OR – odds ratio; 95% CI – confidence interval from two-sided Fisher’s exact test. Values in bold indicate significant differences. Values in parentheses are in percentages. Polymorphism order: rs2248374-rs6861666-rs26653-rs26618-rs2287987-rs30187-rs27044.

**RIF vs. SIVF:** ^a^p/p_corr._ = 0.045/ns, OR = 0.714, 95% CI (0.51-1.00); ^b^p/p_corr._ = 0.040/ns, OR = 1.737, 95% CI (1.02-3.07);

^d^p/p_corr._ = 0.053/ns, OR = Inf., 95% CI (0.83-Inf.);

**All IVF vs. Fertile:** ^c^p/p_corr._ = 0.046/ns, OR = 2.247, 95% CI (0.96-5.84)
