# Supplementary material for: ERAP, KIR, and HLA-C Profile in Recurrent Implantation Failure
Source: Front Immunol. 2021 Oct 22;12:755624. doi: 10.3389/fimmu.2021.755624 (PMC8569704; doi:10.3389/fimmu.2021.755624)
Supplement: Supplementary file 4 [file Table_4.docx]

**Supplementary Table 4** Association between KIR and HLA-C polymorphisms in women participated in IVF-ET and fertile control.

| **KIR/HLA** | **All IVF** | **RIF** | **SIVF** | **Fertile** |
| --- | --- | --- | --- | --- |
|  | N = 196 | N = 106 | N = 66 | N = 162 |
| AA/C1+ | 108 (55.10) | 58 (54.72) | 36 (54.55) | 96 (59.26) |
| AA/C2+ | 88 (44.90) | 48 (45.28) | 30 (45.45) | 66 (40.74) |
|  | N = 137 | N = 77 | N = 44 | N = 110 |
| AA/C1C1 | 49 (35.77) | 29 (37.66) | 14 (31.82) | 44 (40.00) |
| AA/C1C2 | 59 (43.07) | 29 (37.66) | 22 (50.00) | 52 (47.27) |
| AA/C2C2 | 29 (21.16) | **19 (24.68)^a^** | 8 (18.18) | 14 (12.73) |
|  | N = 545 | N = 313 | N = 177 | N = 408 |
| Bx/C1+ | 300 (55.05) | 174 (55.59) | 98 (55.37) | 226 (55.39) |
| Bx/C2+ | 245 (44.95) | 139 (44.41) | 79 (44.63) | 182 (44.61) |
|  | N = 358 | N = 206 | N = 117 | N = 273 |
| Bx/C1C1 | 113 (31.56) | 67 (32.52) | 38 (32.48) | 91 (33.33) |
| Bx/C1C2 | 187 (52.23) | 107 (51.94) | 60 (51.28) | 135 (49.45) |
| Bx/C2C2 | 58 (16.21) | 32 (15.54) | 19 (16.24) | 47 (17.22) |
|  | N = 289 | N = 160 | N = 95 | N = 252 |
| CenAA/C1+ | 160 (55.36) | 89 (55.62) | 52 (54.74) | 147 (58.33) |
| CenAA/C2+ | 129 (44.64) | 71 (44.38) | 43 (45.26) | 105 (41.67) |
|  | N = 196 | N = 112 | N = 61 | N = 172 |
| CenAA/C1C1 | 67 (34.18) | 41 (36.61) | 18 (29.51) | 67 (38.95) |
| CenAA/C1C2 | 93 (47.45) | 48 (42.86) | 34 (55.74) | 80 (46.51) |
| CenAA/C2C2 | 36 (18.37) | 23 (20.53) | 9 (14.75) | 25 (14.54) |
|  | N = 368 | N = 213 | N = 120 | N = 253 |
| CenAB/C1+ | 202 (54.89) | 115 (53.99) | 69 (57.50) | 140 (55.34) |
| CenAB/C2+ | 166 (45.11) | 98 (46.01) | 51 (42.50) | 113 (44.66) |
|  | N = 244 | N = 141 | N = 82 | N = 165 |
| CenAB/C1C1 | 78 (31.97) | 43 (30.50) | 31 (37.80) | 52 (31.52) |
| CenAB/C1C2 | 124 (50.82) | 72 (51.06) | 38 (46.34) | 88 (53.33) |
| CenAB/C2C2 | 42 (17.21) | 26 (18.44) | 13 (15.86) | 25 (15.15) |
|  | N = 84 | N = 46 | N = 28 | N = 65 |
| CenBB/C1+ | 46 (54.76) | 28 (60.87) | 13 (46.43) | 35 (53.85) |
| CenBB/C2+ | 38 (45.24) | 18 (39.13) | 15 (53.57) | 30 (46.15) |
|  | N = 55 | N = 30 | N = 18 | N = 46 |
| CenBB/C1C1 | 17 (30.91) | 12 (40.00) | 3 (16.67) | 16 (34.78) |
| CenBB/C1C2 | 29 (52.73) | 16 (53.33) | 10 (55.56) | 19 (41.30) |
| CenBB/C2C2 | 9 (16.36) | 2 (6.67) | 5 (27.77) | 11 (23.92) |
|  | N = 422 | N = 238 | N = 137 | N = 309 |
| TelAA/C1+ | 229 (54.27) | 128 (53.78) | 74 (54.01) | 182 (58.90) |
| TelAA/C2+ | 193 (45.73) | 110 (46.22) | 63 (45.99) | 127 (41.11) |
|  | N = 285 | N = 164 | N = 90 | N = 204 |
| TelAA/C1C1 | 92 (32.28) | 54 (32.93) | 27 (30.00) | 77 (37.75) |
| TelAA/C1C2 | 137 (48.07) | 74 (45.12) | 47 (52.22) | 105 (51.47) |
| TelAA/C2C2 | **56 (19.65)^b^** | **36 (21.95)^c^** | 16 (17.78) | 22 (10.78) |
|  | N = 268 | N = 157 | N = 87 | N = 225 |
| TelAB/C1+ | 150 (55.97) | 89 (56.69) | 50 (57.47) | 121 (53.78) |
| TelAB/C2+ | 118 (44.03) | 68 (43.31) | 37 (42.53) | 104 (46.22) |
|  | N = 177 | N = 103 | N = 58 | N = 156 |
| TelAB/C1C1 | 59 (33.33) | 35 (33.98) | 21 (36.21) | 52 (33.33) |
| TelAB/C1C2 | 91 (51.41) | 54 (52.43) | 29 (50.00) | 69 (44.23) |
| TelAB/C2C2 | 27 (15.26) | 14 (13.59) | 8 (13.79) | 35 (22.44) |
|  | N = 51 | N = 24 | N = 19 | N = 36 |
| TelBB/C1+ | 29 (56.86) | 15 (62.50) | 10 (52.63) | 19 (52.78) |
| TelBB/C2+ | 22 (43.14) | 9 (37.50) | 9 (47.37) | 17 (47.22) |
|  | N = 33 | N = 16 | N = 13 | N = 23 |
| TelBB/C1C1 | 11 (33.33) | 7 (43.75) | 4 (30.77) | 6 (26.09) |
| TelBB/C1C2 | 18 (54.55) | 8 (50.00) | 6 (46.15) | 13 (56.52) |
| TelBB/C2C2 | 4 (12.12) | 1 (6.25) | 3 (23.08) | 4 (17.39) |
|  | N = 195 | N = 106 | N = 65 | N = 162 |
| CenAA/TelAA/C1+ | 107 (54.87) | 58 (54.72) | 35 (53.85) | 96 (59.26) |
| CenAA/TelAA/C2+ | 88 (45.13) | 48 (45.28) | 30 (46.15) | 66 (40.74) |
|  | N = 136 | N = 77 | N = 43 | N = 110 |
| CenAA/TelAA/C1C1 | 48 (35.29) | 29 (37.66) | 13 (30.23) | 44 (40.00) |
| CenAA/TelAA/C1C2 | 59 (43.38) | 29 (37.66) | 22 (51.16) | 52 (47.27) |
| CenAA/TelAA/C2C2 | 29 (21.33) | **19 (24.68)^d^** | 8 (18.61) | 14 (12.73) |
|  | N = 87 | N = 52 | N = 25 | N = 82 |
| CenAA/TelAB/C1+ | 48 (55.17) | 29 (55.77) | 14 (56.00) | 47 (57.32) |
| CenAA/TelAB/C2+ | 39 (44.83) | 23 (44.23) | 11 (44.00) | 35 (42.68) |
|  | N = 55 | N = 33 | N = 15 | N = 58 |
| CenAA/TelAB/C1C1 | 16 (29.09) | 10 (30.30) | 4 (26.67) | 23 (39.66) |
| CenAA/TelAB/C1C2 | 32 (58.18) | 19 (57.58) | 10 (66.67) | 24 (41.38) |
| CenAA/TelAB/C2C2 | 7 (12.73) | 4 (12.12) | 1 (6.66) | 11 (18.96) |
|  | N = 7 | N = 2 | N = 5 | N = 8 |
| CenAA/TelBB/C1+ | 5 (71.43) | 2 (100.00) | 3 (60.00) | 4 (50.00) |
| CenAA/TelBB/C2+ | 2 (28.57) | 0 (0.00) | 2 (40.00) | 4 (50.00) |
|  | N = 5 | N = 2 | N = 3 | N = 4 |
| CenAA/TelBB/C1C1 | 3 (60.00) | 2 (100.00) | 1 (33.33) | 0 (0.00) |
| CenAA/TelBB/C1C2 | 2 (40.00) | 0 (0.00) | 2 (66.67) | 4 (100.00) |
| CenAA/TelBB/C2C2 | 0 (0.00) | 0 (0.00) | 0 (0.00) | 0 (0.00) |
|  | N = 186 | N = 106 | N = 64 | N = 126 |
| CenAB/TelAA/C1+ | 99 (53.23) | 55 (51.89) | 35 (54.69) | 71 (56.35) |
| CenAB/TelAA/C2+ | 87 (46.77) | 51 (48.11) | 29 (45.31) | 55 (43.65) |
|  | N = 125 | N = 72 | N = 42 | N = 78 |
| CenAB/TelAA/C1C1 | 38 (30.40) | 21 (29.17) | 13 (30.95) | 23 (29.49) |
| CenAB/TelAA/C1C2 | 61 (48.80) | 34 (47.22) | 22 (52.38) | 48 (61.54) |
| CenAB/TelAA/C2C2 | **26 (20.80)^e^** | **17 (23.61)^f^** | 7 (16.67) | 7 (8.97) |
|  | N = 155 | N = 93 | N = 51 | N = 110 |
| CenAB/TelAB/C1+ | 88 (56.77) | 52 (55.91) | 31 (60.78) | 58 (52.73) |
| CenAB/TelAB/C2+ | 67 (43.23) | 41 (44.09) | 20 (39.22) | 52 (47.27) |
|  | N = 102 | N = 60 | N = 36 | N = 76 |
| CenAB/TelAB/C1C1 | 35 (34.31) | 19 (31.67) | 16 (44.44) | 24 (31.58) |
| CenAB/TelAB/C1C2 | 53 (51.96) | 33 (55.00) | 15 (41.67) | 34 (44.74) |
| CenAB/TelAB/C2C2 | 14 (13.73) | 8 (13.33) | 5 (13.89) | 18 (23.68) |
|  | N = 27 | N = 14 | N = 5 | N = 17 |
| CenAB/TelBB/C1+ | 15 (55.56) | 8 (57.14) | 3 (60.00) | 11 (64.71) |
| CenAB/TelBB/C2+ | 12 (44.44) | 6 (42.86) | 2 (40.00) | 6 (35.29) |
|  | N = 17 | N = 9 | N = 4 | N = 11 |
| CenAB/TelBB/C1C1 | 5 (29.41) | 3 (33.33) | 2 (50.00) | 5 (45.45) |
| CenAB/TelBB/C1C2 | 10 (58.82) | 5 (55.56) | 1 (25.00) | 6 (54.55) |
| CenAB/TelBB/C2C2 | 2 (11.77) | 1 (11.11) | 1 (25.00) | 0 (0.00) |
|  | N = 41 | N = 26 | N = 8 | N = 21 |
| CenBB/TelAA/C1+ | 23 (56.10) | 15 (57.69) | 4 (50.00) | 15 (71.43) |
| CenBB/TelAA/C2+ | 18 (43.90) | 11 (42.31) | 4 (50.00) | 6 (28.57) |
|  | N = 24 | N = 15 | N = 5 | N = 16 |
| CenBB/TelAA/C1C1 | **6 (25.00)^g^** | 4 (26.67) | 1 (20.00) | 10 (62.50) |
| CenBB/TelAA/C1C2 | **17 (70.83)^h^** | **11 (73.33)^i^** | 3 (60.00) | 5 (31.25) |
| CenBB/TelAA/C2C2 | 1 (4.17) | 0 (0.00) | 1 (20.00) | 1 (6.25) |
|  | N = 26 | N = 12 | N = 11 | N = 33 |
| CenBB/TelAB/C1+ | 14 (53.85) | 8 (66.67) | 5 (45.45) | 16 (48.48) |
| CenBB/TelAB/C2+ | 12 (46.15) | 4 (33.33) | 6 (54.55) | 17 (51.52) |
|  | N = 20 | N = 10 | N = 7 | N = 22 |
| CenBB/TelAB/C1C1 | 8 (40.00) | 6 (60.00) | 1 (14.29) | 5 (22.73) |
| CenBB/TelAB/C1C2 | 6 (30.00) | 2 (20.00) | 4 (57.14) | 11 (50.00) |
| CenBB/TelAB/C2C2 | 6 (30.00) | 2 (20.00) | 2 (28.57) | 6 (27.27) |
|  | N = 17 | N = 8 | N = 9 | N = 11 |
| CenBB/TelBB/C1+ | 9 (52.94) | 5 (62.50) | 4 (44.44) | 4 (36.36) |
| CenBB/TelBB/C2+ | 8 (47.06) | 3 (37.50) | 5 (55.56) | 7 (63.64) |
|  | N = 11 | N = 5 | N = 6 | N = 8 |
| CenBB/TelBB/C1C1 | 3 (27.27) | 2 (40.00) | 1 (16.67) | 1 (12.50) |
| CenBB/TelBB/C1C2 | 6 (54.55) | 3 (60.00) | 3 (50.00) | 3 (37.50) |
| CenBB/TelBB/C2C2 | 2 (18.18) | 0 (0.00) | 2 (33.33) | 4 (50.00) |

IVF-ET – in vitro fertilization embryo transfer; RIF – recurrent implantation failure; SIVF – successful pregnancy after IVF-ET; p – probability; p_corr._ – probability after Bonferroni correction for multiple comparisons (x 3 for HLA-C possible combinations); OR – odds ratio; 95% CI – confidence interval from two-sided Fisher’s exact test; ns – not significant. Values in bold indicate significant differences. Values in parentheses are in percentages.

**RIF vs Fertile:** ^a^p/p_corr._ = 0.050/ns, OR = 2.236, 95% CI (0.98-5.22); ^c^p/p_corr._ = 0.004/0.012, OR = 2.321, 95% CI (1.26-4.35); ^d^p/p_corr._ = 0.050/ns, OR = 2.236, 95% CI (0.98-5.22); ^f^p/p_corr._ = 0.024/ns, OR = 3.111, 95% CI (1.13-9.53); ^i^p/p_corr._ = 0.032/ns, OR = 5.663, 95% CI (1.03-38.63);

**All vs Fertile:** ^b^p/p_corr._ = 0.009/0.026, OR = 2.020, 95% CI (1.16-3.61); ^e^p/p_corr._ = 0.031/ns, OR = 2.652, 95% CI (1.05-7.65); ^g^p/p_corr._ = 0.025/ns, OR = 0.209, 95% CI (0.04-0.95); ^h^p/p_corr._ = 0.023/ns, OR = 5.097, 95% CI (1.13-26.76)
