# Supplementary material for: ERAP, KIR, and HLA-C Profile in Recurrent Implantation Failure
Source: Front Immunol. 2021 Oct 22;12:755624. doi: 10.3389/fimmu.2021.755624 (PMC8569704; doi:10.3389/fimmu.2021.755624)
Supplement: Supplementary file 5 [file Table_5.docx]

**Supplementary Table 5** Association between ERAP and HLA-C polymorphisms in women participated in IVF-ET and fertile control.

| **ERAP/HLA-C** | **All IVF** | **RIF** | **SIVF** | **Fertile** |
| --- | --- | --- | --- | --- |
| **ERAP1 rs30187/HLA-C** | N = 408 | N = 232 | N = 134 | N = 322 |
| CC/C1+ | 189 (46.32) | 102 (43.97) | 63 (47.01) | 153 (47.52) |
| CT/C1+ | 176 (43.14) | 105 (45.26) | 57 (42.54) | 139 (43.17) |
| TT/C1+ | 43 (10.54) | 25 (10.77) | 14 (10.45) | 30 (9.31) |
|  | N = 333 | N = 187 | N = 109 | N = 248 |
| CC/C2+ | 148 (44.44) | 77 (41.18) | 50 (45.87) | 118 (47.58) |
| CT/C2+ | 149 (44.74) | 85 (45.45) | 51 (46.79) | 114 (45.97) |
| TT/C2+ | 36 (10.82) | **25 (13.37)^a^** | 8 (7.34) | 16 (6.45) |
|  | N = 162 | N = 96 | N = 52 | N = 135 |
| CC/C1C1 | 73 (45.06) | 41 (42.71) | 25 (48.08) | 66 (48.89) |
| CT/C1C1 | 69 (42.59) | 45 (46.88) | 19 (36.54) | 51 (37.78) |
| TT/C1C1 | 20 (12.35) | 10 (10.41) | 8 (15.38) | 18 (13.33) |
|  | N = 246 | N = 136 | N = 82 | N = 187 |
| CC/C1C2 | 116 (47.15) | 61 (44.85) | 38 (46.34) | 87 (46.52) |
| CT/C1C2 | 107 (43.50) | 60 (44.12) | 38 (46.34) | 88 (47.06) |
| TT/C1C2 | 23 (9.35) | 15 (11.03) | 6 (7.32) | 12 (6.42) |
|  | N = 87 | N = 51 | N = 27 | N = 61 |
| CC/C2C2 | 32 (36.78) | **16 (31.37)^b^** | 12 (44.44) | 31 (50.82) |
| CT/C2C2 | 42 (48.28) | 25 (49.02) | 13 (48.15) | 26 (42.62) |
| TT/C2C2 | 13 (14.94) | **10 (19.61)^c^** | 2 (7.41) | 4 (6.56) |
| **ERAP1 rs27044/HLA-C** | N = 408 | N = 232 | N = 134 | N = 322 |
| CC/C1+ | 224 (54.90) | 120 (51.72) | 74 (55.22) | 189 (58.70) |
| CG/C1+ | 157 (38.48) | 95 (40.95) | 52 (38.81) | 109 (33.85) |
| GG/C1+ | 27 (6.62) | 17 (7.33) | 8 (5.97) | 24 (7.45) |
|  | N = 333 | N = 187 | N = 109 | N = 248 |
| CC/C2+ | 181 (54.35) | 96 (51.34) | 58 (53.21) | 143 (57.66) |
| CG/C2+ | 131 (39.34) | 77 (41.18) | 46 (42.20) | 94 (37.90) |
| GG/C2+ | 21 (6.31) | 14 (7.48) | 5 (4.59) | 11 (4.44) |
|  | N = 162 | N = 96 | N = 52 | N = 135 |
| CC/C1C1 | 83 (51.23) | 45 (46.88) | 30 (57.69) | 81 (60.00) |
| CG/C1C1 | **65 (40.12)^d^** | **43 (44.79)^e^** | 17 (32.69) | 38 (28.15) |
| GG/C1C1 | 14 (8.65) | 8 (8.33) | 5 (9.61) | 16 (11.85) |
|  | N = 246 | N = 136 | N = 82 | N = 187 |
| CC/C1C2 | 141 (57.32) | 75 (55.15) | 44 (53.66) | 108 (57.75) |
| CG/C1C2 | 92 (37.40) | 52 (38.24) | 35 (42.68) | 71 (37.97) |
| GG/C1C2 | 13 (5.28) | 9 (6.61) | 3 (3.66) | 8 (4.28) |
|  | N = 87 | N = 51 | N = 27 | N = 61 |
| CC/C2C2 | 40 (45.98) | 21 (41.18) | 14 (51.85) | 35 (57.38) |
| CG/C2C2 | 39 (44.82) | 25 (49.02) | 11 (40.74) | 23 (37.70) |
| GG/C2C2 | 8 (9.20) | 5 (9.80) | 2 (7.41) | 3 (4.92) |
| **ERAP1 rs26653/HLA-C** | N = 408 | N = 232 | N = 134 | N = 320 |
| GG/C1+ | 231 (56.62) | 130 (56.03) | 74 (55.22) | 169 (52.81) |
| CG/C1+ | 157 (38.48) | 91 (39.22) | 56 (41.79) | 134 (41.88) |
| CC/C1+ | 20 (4.90) | 11 (4.75) | 4 (2.99) | 17 (5.31) |
|  | N = 333 | N = 187 | N = 109 | N = 246 |
| GG/C2+ | 182 (54.65) | 98 (52.41) | 62 (56.88) | 140 (56.91) |
| CG/C2+ | 136 (40.84) | 80 (42.78) | 43 (39.45) | 96 (39.02) |
| CC/C2+ | 15 (4.51) | 9 (4.81) | 4 (3.67) | 10 (4.07) |
|  | N = 162 | N = 96 | N = 52 | N = 135 |
| GG/C1C1 | 88 (54.32) | 51 (53.12) | 27 (51.92) | 72 (53.33) |
| CG/C1C1 | 64 (39.51) | 40 (41.67) | 23 (44.23) | 54 (40.00) |
| CC/C1C1 | 10 (6.17) | 5 (5.21) | 2 (3.85) | 9 (6.67) |
|  | N = 246 | N = 136 | N = 82 | N = 185 |
| GG/C1C2 | 143 (58.13) | 79 (58.09) | 47 (57.32) | 97 (52.43) |
| CG/C1C2 | 93 (37.80) | 51 (37.50) | 33 (40.24) | 80 (43.24) |
| CC/C1C2 | 10 (4.07) | 6 (4.41) | 2 (2.44) | 8 (4.33) |
|  | N = 87 | N = 51 | N = 27 | N = 61 |
| GG/C2C2 | **39 (44.83)^f^** | **19 (37.25)^g^** | 15 (55.56) | 43 (70.49) |
| CG/C2C2 | **43 (49.43)^h^** | **29 (56.86)^i^** | 10 (37.04) | 16 (26.23) |
| CC/C2C2 | 5 (5.74) | 3 (5.89) | 2 (7.40) | 2 (3.28) |
| **ERAP1 rs26618/HLA-C** | N = 408 | N = 232 | N = 134 | N = 322 |
| TT/C1+ | 212 (51.96) | 129 (55.60) | 65 (48.51) | 161 (50.00) |
| CT/C1+ | 163 (39.95) | 84 (36.21) | 57 (42.54) | 135 (41.93) |
| CC/C1+ | 33 (8.09) | 19 (8.19) | 12 (8.95) | 26 (8.07) |
|  | N = 333 | N = 187 | N = 109 | N = 248 |
| TT/C2+ | 186 (55.86) | **116 (62.03) ^j,k^** | 54 (49.54) | 120 (48.39) |
| CT/C2+ | 123 (36.94) | **62 (33.16)^l^** | 44 (40.37) | 109 (43.95) |
| CC/C2+ | 24 (7.20) | 9 (4.81) | 11 (10.09) | 19 (7.66) |
|  | N = 162 | N = 96 | N = 52 | N = 135 |
| TT/C1C1 | 81 (50.00) | 47 (48.96) | 27 (51.92) | 70 (51.85) |
| CT/C1C1 | 65 (40.12) | 37 (38.54) | 21 (40.38) | 52 (38.52) |
| CC/C1C1 | 16 (9.88) | 12 (12.50) | 4 (7.70) | 13 (9.63) |
|  | N = 246 | N = 136 | N = 82 | N = 187 |
| TT/C1C2 | 131 (53.25) | **82 (60.29)^m,n^** | 38 (46.34) | 91 (48.66) |
| CT/C1C2 | 98 (39.84) | 47 (34.56) | 36 (43.90) | 83 (44.39) |
| CC/C1C2 | 17 (6.91) | 7 (5.15) | 8 (9.76) | 13 (6.95) |
|  | N = 87 | N = 51 | N = 27 | N = 61 |
| TT/C2C2 | 55 (63.22) | 34 (66.67) | 16 (59.26) | 29 (47.54) |
| CT/C2C2 | 25 (28.74) | 15 (29.41) | 8 (29.63) | 26 (42.62) |
| CC/C2C2 | 7 (8.04) | 2 (3.92) | 3 (11.11) | 6 (9.84) |
| **ERAP1 rs2287987/HLA-C** | N = 408 | N = 232 | N = 134 | N = 322 |
| TT/C1+ | 248 (60.78) | 140 (60.34) | 87 (64.93) | 192 (59.63) |
| CT/C1+ | 138 (33.82) | 80 (34.48) | 39 (29.1) | 121 (37.58) |
| CC/C1+ | 22 (5.40) | 12 (5.18) | 8 (5.96) | 9 (2.9) |
|  | N = 333 | N = 187 | N = 109 | N = 248 |
| TT/C2+ | 203 (60.96) | 111 (59.36) | 70 (64.22) | 153 (61.69) |
| CT/C2+ | 111 (33.33) | 65 (34.76) | 33 (30.28) | 87 (35.08) |
| CC/C2+ | 19 (5.71) | 11 (5.88) | 6 (5.50) | 8 (3.23) |
|  | N = 162 | N = 96 | N = 52 | N = 135 |
| TT/C1C1 | 103 (63.58) | 64 (66.67) | 33 (63.46) | 73 (54.07) |
| CT/C1C1 | 54 (33.33) | **29 (30.21)^o^** | 17 (32.69) | 59 (43.70) |
| CC/C1C1 | 5 (3.09) | 3 (3.12) | 2 (3.85) | 3 (2.23) |
|  | N = 246 | N = 136 | N = 82 | N = 187 |
| TT/C1C2 | 145 (58.94) | 76 (55.88) | 54 (65.85) | 119 (63.64) |
| CT/C1C2 | 84 (34.15) | 51 (37.50) | 22 (26.83) | 62 (33.16) |
| CC/C1C2 | 17 (6.91) | 9 (6.62) | 6 (7.32) | 6 (3.20) |
|  | N = 87 | N = 51 | N = 27 | N = 61 |
| TT/C2C2 | 58 (66.67) | 35 (68.63) | 16 (59.26) | 34 (55.74) |
| CT/C2C2 | 27 (31.03) | 14 (27.45) | 11 (40.74) | 25 (40.98) |
| CC/C2C2 | 2 (2.30) | 2 (3.92) | 0 (0.00) | 2 (3.28) |
| **ERAP2 rs2248374/HLA-C** | N = 407 | N = 231 | N = 134 | N = 320 |
| AA/C1+ | 114 (28.01) | 61 (26.41) | 44 (32.84) | 91 (28.44) |
| AG/C1+ | 193 (47.42) | 107 (46.32) | 64 (47.76) | 156 (48.75) |
| GG/C1+ | 100 (24.57) | 63 (27.27) | 26 (19.40) | 73 (22.81) |
|  | N = 332 | N = 186 | N = 109 | N = 246 |
| AA/C2+ | 78 (23.49) | 41 (22.04) | 30 (27.52) | 67 (27.24) |
| AG/C2+ | 166 (50.00) | 94 (50.54) | 52 (47.71) | 118 (47.97) |
| GG/C2+ | 88 (26.51) | 51 (27.42) | 27 (24.77) | 61 (24.9) |
|  | N = 162 | N = 96 | N = 52 | N = 135 |
| AA/C1C1 | 48 (29.63) | 26 (27.08) | 18 (34.62) | 38 (28.15) |
| AG/C1C1 | 71 (43.83) | 40 (41.67) | 24 (46.15) | 63 (46.67) |
| GG/C1C1 | 43 (26.54) | 30 (31.25) | 10 (19.23) | 34 (25.18) |
|  | N = 245 | N = 135 | N = 82 | N = 185 |
| AA/C1C2 | 66 (26.94) | 35 (25.93) | 26 (31.71) | 53 (28.65) |
| AG/C1C2 | 122 (49.80) | 67 (49.63) | 40 (48.78) | 93 (50.27) |
| GG/C1C2 | 57 (23.26) | 33 (24.44) | 16 (19.51) | 39 (21.08) |
|  | N = 87 | N = 51 | N = 27 | N = 61 |
| AA/C2C2 | 12 (13.79) | 6 (11.77) | 4 (14.82) | 14 (22.95) |
| AG/C2C2 | 44 (50.57) | 27 (52.94) | 12 (44.44) | 25 (40.98) |
| GG/C2C2 | 31 (35.64) | 18 (35.29) | 11 (40.74) | 22 (36.07) |
| **ERAP1 rs6861666/HLA-C** | N = 407 | N = 231 | N = 134 | N = 319 |
| AA/C1+ | 349 (85.75) | 194 (83.98) | 116 (86.57) | 280 (87.77) |
| AG/C1+ | 57 (14.00) | 36 (15.58) | 18 (13.43) | 39 (12.23) |
| GG/C1+ | 1 (0.25) | 1 (0.44) | 0 (0.00) | 0 (0.00) |
|  | N = 332 | N = 186 | N = 109 | N = 244 |
| AA/C2+ | 289 (87.05) | 159 (85.48) | 95 (87.16) | 207 (84.84) |
| AG/C2+ | 41 (12.35) | 26 (13.98) | 13 (11.93) | 36 (14.75) |
| GG/C2+ | 2 (0.60) | 1 (0.54) | 1 (0.91) | 1 (0.41) |
|  | N = 162 | N = 96 | N = 52 | N = 135 |
| AA/C1C1 | 134 (82.72) | 79 (82.29) | 43 (82.69) | 118 (87.41) |
| AG/C1C1 | 28 (17.28) | 17 (17.71) | 9 (17.31) | 17 (12.59) |
| GG/C1C1 | 0 (0.00) | 0 (0.00) | 0 (0.00) | 0 (0.00) |
|  | N = 245 | N = 135 | N = 82 | N = 184 |
| AA/C1C2 | 215 (87.76) | 115 (85.19) | 73 (89.02) | 162 (88.04) |
| AG/C1C2 | 29 (11.84) | 19 (14.07) | 9 (10.98) | 22 (11.96) |
| GG/C1C2 | 1 (0.40) | 1 (0.74) | 0 (0.00) | 0 (0.00) |
|  | N = 87 | N = 51 | N = 27 | N = 60 |
| AA/C2C2 | 74 (85.06) | 44 (86.27) | 22 (81.48) | 45 (75.00) |
| AG/C2C2 | 12 (13.79) | 7 (13.73) | 4 (14.81) | 14 (23.33) |
| GG/C2C2 | 1 (1.15) | 0 (0.00) | 1 (3.71) | 1 (1.67) |

IVF-ET – in vitro fertilization embryo transfer; RIF – recurrent implantation failure; SIVF – successful pregnancy after IVF-ET; p – probability; p_corr._ – probability after Bonferroni correction for multiple comparisons (x 6 for HLA-C alleles or x 9 for HLA-C genotypes); OR – odds ratio; 95% CI – confidence interval from two-sided Fisher’s exact test; ns – not significant. Values in bold indicate significant differences. Values in parentheses are in percentages.

**RIF vs. Fertile:** ^a^p/p_corr._ = 0.020/ns, OR = 2.233, 95% CI (1.11-4.63); ^b^p/p_corr._ = 0.054/ns, OR = 0.446, 95% CI (0.19-1.03); ^c^p/p_corr._ = 0.047/ns, OR = 3.437, 95% CI (0.91-16.07); ^e^p/p_corr._ = 0.012/ns, OR = 2.064, 95% CI (1.15-3.72); ^g^p/p_corr._ = 0.001/0.005, OR = 0.252, 95% CI (0.10-0.59); ^i^p/p_corr._ = 0.002/0.016, OR = 3.661, 95% CI (1.56-8.89); ^j^p/p_corr._ = 0.005/0.029, OR = 1.741, 95% CI (1.16-2.61); ^l^p/p_corr._ = 0.023/ns, OR = 0.633, 95% CI (0.42-0.96); ^m^p/p_corr._ = 0.042/ns, OR = 1.600, 95% CI (1.00-2.57); ^o^p/p_corr._ = 0.040/ns, OR = 0.559, 95% CI (0.31-1.00);

**All IVF vs. Fertile:** ^d^p/p_corr._ = 0.037/ns, OR = 1.707, 95% CI (1.02-2.88); ^f^p/p_corr._ = 0.002/0.022, OR = 0.343, 95% CI (0.16-0.72); ^h^p/p_corr._ = 0.006/ns, OR = 2.730, 95% CI (1.29-6.00);

**RIF vs. SIVF:** ^k^p/p_corr._ = 0.039/ns, OR = 1.661, 95% CI (1.00-2.76); ^n^p/p_corr._ = 0.050/ns, OR = 1.754, 95% CI (0.97-3.18)
