# Supplementary material for: ERAP, KIR, and HLA-C Profile in Recurrent Implantation Failure
Source: Front Immunol. 2021 Oct 22;12:755624. doi: 10.3389/fimmu.2021.755624 (PMC8569704; doi:10.3389/fimmu.2021.755624)
Supplement: Supplementary file 6 [file Table_6.docx]

**Supplementary Table 6** Association between ERAP1 rs26618 and KIR polymorphism in women participated in IVF-ET and fertile control.

| **ERAP1 rs26618/KIR** | **All IVF** | **RIF** | **SIVF** | **Fertile** |
| --- | --- | --- | --- | --- |
|  | N = 138 | N = 77 | N = 44 | N = 110 |
| TT/AA+ | 79 (57.25) | 44 (57.14) | 26 (59.09) | 51 (46.36) |
| CT/AA+ | 53 (38.41) | 28 (36.36) | 17 (38.64) | 44 (40.00) |
| CC/AA+ | **6 (4.34)^a^** | 5 (6.50) | **1 (2.27)^b^** | 15 (13.64) |
|  | N = 358 | N = 206 | N = 117 | N = 275 |
| TT/Bx+ | 189 (52.79) | 119 (57.77) | 55 (47.01) | 139 (50.55) |
| CT/Bx+ | 135 (37.71) | 71 (34.47) | 48 (41.03) | 118 (42.91) |
| CC/Bx+ | 34 (9.50) | 16 (7.76) | 14 (11.96) | 18 (6.54) |
|  | N = 197 | N = 112 | N = 61 | N = 173 |
| TT/cenAA | **114 (57.87)^c^** | **68 (60.71)^d^** | 34 (55.74) | 80 (46.24) |
| CT/cenAA | 72 (36.55) | 37 (33.04) | 24 (39.34) | 76 (43.93) |
| CC/cenAA | 11 (5.58) | 7 (6.25) | 3 (4.92) | 17 (9.83) |
|  | N = 244 | N = 141 | N = 82 | N = 166 |
| TT/cenAB | 137 (56.15) | **88 (62.41)^e,f^** | 40 (48.78) | 84 (50.60) |
| CT/cenAB | 83 (34.02) | **42 (29.79)^g^** | 32 (39.02) | 69 (41.57) |
| CC/cenAB | 24 (9.83) | 11 (7.80) | 10 (12.20) | 13 (7.83) |
|  | N = 55 | N = 30 | N = 18 | N = 46 |
| TT/cenBB | **17 (30.91)^h^** | **7 (23.33)^i^** | 7 (38.89) | 26 (56.52) |
| CT/cenBB | **33 (60.00)^j^** | **20 (66.67)^k^** | 9 (50.00) | 17 (36.96) |
| CC/cenBB | 5 (9.09) | 3 (10.00) | 2 (11.11) | 3 (6.52) |
|  | N = 286 | N = 164 | N = 90 | N = 205 |
| TT/telAA | 150 (52.45) | 88 (53.66) | 48 (53.33) | 93 (45.37) |
| CT/telAA | 115 (40.21) | 64 (39.02) | 34 (37.78) | 89 (43.41) |
| CC/telAA | 21 (7.33) | 12 (7.32) | 8 (8.89) | 23 (11.22) |
|  | N = 177 | N = 103 | N = 58 | N = 156 |
| TT/telAB | 102 (57.63) | 64 (62.14) | 29 (50.00) | 81 (51.92) |
| CT/telAB | 58 (32.77) | **30 (29.13)^l^** | 23 (39.66) | 66 (42.31) |
| CC/telAB | 17 (9.60) | 9 (8.73) | 6 (10.34) | 9 (5.77) |
|  | N = 33 | N = 16 | N = 13 | N = 23 |
| TT/telBB | 16 (48.48) | 11 (68.75) | **4 (30.77)^m^** | 16 (69.57) |
| CT/telBB | 15 (45.45) | 5 (31.25) | 8 (61.54) | 6 (26.09) |
| CC/telBB | 2 (6.06) | 0 (0.00) | 1 (7.69) | 1 (4.34) |
|  | N = 137 | N = 77 | N = 43 | N = 110 |
| TT/cenAA/telAA | 78 (56.93) | 44 (57.14) | 25 (58.14) | 51 (46.36) |
| CT/cenAA/telAA | 53 (38.69) | 28 (36.36) | 17 (39.53) | 44 (40.00) |
| CC/cenAA/telAA | **6 (4.38)^n^** | 5 (6.50) | **1 (2.33)^o^** | 15 (13.64) |
|  | N = 55 | N = 33 | N = 15 | N = 58 |
| TT/cenAA/telAB | 34 (61.82) | **23 (69.70)^p^** | 8 (53.33) | 25 (43.10) |
| CT/cenAA/telAB | **17 (30.91)^q^** | **8 (24.24)^r^** | 6 (40.00) | 31 (53.45) |
| CC/cenAA/telAB | 4 (7.27) | 2 (6.06) | 1 (6.67) | 2 (3.45) |
|  | N = 5 | N = 2 | N = 3 | N = 4 |
| TT/cenAA/telBB | 2 (40.00) | 1 (50.00) | 1 (33.33) | 4 (100.00) |
| CT/cenAA/telBB | 2 (40.00) | 1 (50.00) | 1 (33.33) | 0 (0.00) |
| CC/cenAA/telBB | 1 (20.00) | 0 (0.00) | 1 (33.33) | 0 (0.00) |
|  | N = 125 | N = 72 | N = 42 | N = 79 |
| TT/cenAB/telAA | 65 (52.00) | 40 (55.56) | 21 (50.00) | 33 (41.77) |
| CT/cenAB/telAA | 48 (38.40) | 26 (36.11) | 16 (38.10) | 38 (48.10) |
| CC/cenAB/telAA | 12 (9.60) | 6 (8.33) | 5 (11.90) | 8 (10.13) |
|  | N = 102 | N = 60 | N = 36 | N = 76 |
| TT/cenAB/telAB | 63 (61.76) | 41 (68.33) | 18 (50.00) | 45 (59.21) |
| CT/cenAB/telAB | 28 (27.45) | 14 (23.33) | 13 (36.11) | 26 (34.21) |
| CC/cenAB/telAB | 11 (10.79) | 5 (8.34) | 5 (13.89) | 5 (6.58) |
|  | N = 17 | N = 9 | N = 4 | N = 11 |
| TT/cenAB/telBB | 9 (52.94) | 7 (77.78) | 1 (25.00) | 6 (54.55) |
| CT/cenAB/telBB | 7 (41.18) | 2 (22.22) | 3 (75.00) | 5 (45.45) |
| CC/cenAB/telBB | 1 (5.88) | 0 (0.00) | 0 (0.00) | 0 (0.00) |
|  | N = 24 | N = 15 | N = 5 | N = 16 |
| TT/cenBB/telAA | 7 (29.17) | 4 (26.67) | 2 (40.00) | 9 (56.25) |
| CT/cenBB/telAA | 14 (58.33) | 10 (66.67) | 1 (20.00) | 7 (43.75) |
| CC/cenBB/telAA | 3 (12.50) | 1 (6.66) | **2 (40.00)^s^** | 0 (0.00) |
|  | N = 20 | N = 10 | N = 7 | N = 22 |
| TT/cenBB/telAB | 5 (25.00) | **0 (0.00)^t,u^** | 3 (42.86) | 11 (50.00) |
| CT/cenBB/telAB | 13 (65.00) | 8 (80.00) | 4 (57.14) | 9 (40.91) |
| CC/cenBB/telAB | 2 (10.00) | 2 (20.00) | 0 (0.00) | 2 (9.09) |
|  | N = 11 | N = 5 | N = 6 | N = 8 |
| TT/cenBB/telBB | 5 (45.45) | 3 (60.00) | 2 (33.33) | 6 (75.00) |
| CT/cenBB/telBB | 6 (54.55) | 2 (40.00) | 4 (66.67) | 1 (12.50) |
| CC/cenBB/telBB | 0 (0.00) | 0 (0.00) | 0 (0.00) | 1 (12.50) |

IVF-ET – in vitro fertilization embryo transfer; RIF – recurrent implantation failure; SIVF – successful pregnancy after IVF-ET; p – probability; p_corr._ – probability after Bonferroni correction for multiple comparisons (x 6 for AA+/Bx combinations; x 9 for KIR centromeric or telomeric combinations; x 27 for KIR centromeric and telomeric combiantions); OR – odds ratio; 95% CI – confidence interval from two-sided Fisher’s exact test; ns – not significant. Values in bold indicate significant differences. Values in parentheses are in percentages.

**All IVF vs. Fertile:** ^a^p/p_corr._ = 0.011/ns, OR = 0.289, 95% CI (0.09-0.82); ^c^p/p_corr._ = 0.029/ns, OR = 1.595, 95% CI (1.04-2.46); ^h^p/p_corr._ = 0.015/ns, OR = 0.348, 95% CI (0.14-0.84); ^j^p/p_corr._ = 0.028/ns, OR = 2.534, 95% CI (1.06-6.21); ^n^p/p_corr._ = 0.011/ns, OR = 0.292, 95% CI (0.09-0.83); ^q^p/p_corr._ = 0.022/ns, OR = 0.393, 95% CI (0.17-0.90);

**SIVF vs. Fertile:** ^b^p/p_corr._ = 0.041/ns, OR = 0.148, 95% CI (0.00-1.03); ^m^p/p_corr._ = 0.038/ns, OR = 0.205, 95% CI (0.03-1.04); ^o^p/p_corr._ = 0.042/ns, OR = 0.152, 95% CI (0.00-1.05); ^s^p/p_corr._ = 0.048/ns, OR = Inf., 95% CI (0.67-Inf.);

**RIF vs. Fertile:** ^d^p/p_corr._ = 0.021/ns, OR = 1.793, 95% CI (1.08-3.00); ^e^p/p_corr._ = 0.039/ns, OR = 1.618, 95% CI (1.00-2.63); ^g^p/p_corr._ = 0.043/ns, OR = 0.597, 95% CI (0.36-0.98); ^i^p/p_corr._ = 0.005/0.045, OR = 0.239, 95% CI (0.07-0.72); ^k^p/p_corr._ = 0.018/ns, OR = 3.354, 95% CI (1.18-10.12); ^l^p/p_corr._ = 0.036/ns, OR = 0.562, 95% CI (0.32-0.98); ^p^p/p_corr._ = 0.017/ns, OR = 2.998, 95% CI (1.13-8.43); ^r^p/p_corr._ = 0.008/ns, OR = 0.283, 95% CI (0.09-0.78); ^t^p/p_corr._ = 0.006/ns, OR = 0.000, 95% CI (0.00-0.61);

**RIF vs. SIVF:** ^f^p/p_corr._ = 0.051/ns, OR = 1.739, 95% CI (0.97-3.14); ^u^p/p_corr._ = 0.051, OR = 0.000, 95% CI (0.00-1.47)
