# Supplementary material for: ERAP, KIR, and HLA-C Profile in Recurrent Implantation Failure
Source: Front Immunol. 2021 Oct 22;12:755624. doi: 10.3389/fimmu.2021.755624 (PMC8569704; doi:10.3389/fimmu.2021.755624)
Supplement: Supplementary file 7 [file Table_7.docx]

**Supplementary Table 7** Association between ERAP1 rs26653 and KIR polymorphism in women participated in IVF-ET and fertile control.

| **ERAP1 rs26653/KIR** | **All IVF** | **RIF** | **SIVF** | **Fertile** |
| --- | --- | --- | --- | --- |
|  | N = 138 | N = 77 | N = 44 | N = 109 |
| GG/AA+ | 71 (51.45) | 37 (48.05) | 23 (52.27) | 65 (59.63) |
| CG/AA+ | 56 (40.58) | 35 (45.45) | 16 (36.36) | 39 (35.78) |
| CC/AA+ | 11 (7.97) | 5 (6.50) | 5 (11.37) | 5 (4.59) |
|  | N = 358 | N = 206 | N = 117 | N = 273 |
| GG/Bx+ | 199 (55.59) | 112 (54.37) | 66 (56.41) | 148 (54.21) |
| CG/Bx+ | 145 (40.50) | 85 (41.26) | 50 (42.74) | 111 (40.66) |
| CC/Bx+ | 14 (3.91) | 9 (4.37) | **1 (0.85)^a^** | 14 (5.13) |
|  | N = 197 | N = 112 | N = 61 | N = 171 |
| GG/cenAA | 103 (52.28) | 55 (49.11) | 32 (52.46) | 100 (58.48) |
| CG/cenAA | 78 (39.59) | 48 (42.86) | 24 (39.34) | 65 (38.01) |
| CC/cenAA | 16 (8.13) | 9 (8.03) | 5 (8.20) | 6 (3.51) |
|  | N = 244 | N = 141 | N = 82 | N = 165 |
| GG/cenAB | 129 (52.87) | 72 (51.06) | 45 (54.88) | 90 (54.55) |
| CG/cenAB | 106 (43.44) | 64 (45.39) | 36 (43.90) | 65 (39.39) |
| CC/cenAB | 9 (3.69) | 5 (3.55) | 1 (1.22) | 10 (6.06) |
|  | N = 55 | N = 30 | N = 18 | N = 46 |
| GG/cenBB | 38 (69.09) | 22 (73.33) | 12 (66.67) | 23 (50.00) |
| CG/cenBB | 17 (30.91) | 8 (26.67) | 6 (33.33) | 20 (43.48) |
| CC/cenBB | 0 (0.00) | 0 (0.00) | 0 (0.00) | 3 (6.52) |
|  | N = 286 | N = 164 | N = 90 | N = 203 |
| GG/telAA | 156 (54.55) | 88 (53.66) | 47 (52.22) | 116 (57.14) |
| CG/telAA | 114 (39.86) | 68 (41.46) | 38 (42.22) | 75 (36.95) |
| CC/telAA | 16 (5.59) | 8 (4.88) | 5 (5.56) | 12 (5.91) |
|  | N = 177 | N = 103 | N = 58 | N = 156 |
| GG/telAB | 98 (55.37) | 58 (56.31) | 31 (53.45) | 86 (55.13) |
| CG/telAB | 72 (40.68) | 40 (38.83) | 26 (44.83) | 64 (41.03) |
| CC/telAB | 7 (3.95) | 5 (4.86) | 1 (1.72) | 6 (3.84) |
|  | N = 33 | N = 16 | N = 13 | N = 23 |
| GG/telBB | 16 (48.48) | **3 (18.75)^b^** | **11 (84.62)^c^** | 11 (47.83) |
| CG/telBB | 15 (45.45) | **12 (75.00)^d^** | 2 (15.38) | 11 (47.83) |
| CC/telBB | 2 (6.07) | 1 (6.25) | 0 (0.00) | 1 (4.34) |
|  | N = 137 | N = 77 | N = 43 | N = 109 |
| GG/cenAA/telAA | 70 (51.09) | 37 (48.05) | 22 (51.16) | 65 (59.63) |
| CG/cenAA/telAA | 56 (40.88) | 35 (45.45) | 16 (37.21) | 39 (35.78) |
| CC/cenAA/telAA | 11 (8.03) | 5 (6.50) | 5 (11.63) | 5 (4.59) |
|  | N = 55 | N = 33 | N = 15 | N = 58 |
| GG/cenAA/telAB | 30 (54.55) | 18 (54.55) | 7 (46.67) | 35 (60.34) |
| CG/cenAA/telAB | 21 (38.18) | 12 (36.36) | 8 (53.33) | 22 (37.93) |
| CC/cenAA/telAB | 4 (7.27) | 3 (9.09) | 0 (0.00) | 1 (1.73) |
|  | N = 5 | N = 2 | N = 3 | N = 4 |
| GG/cenAA/telBB | 3 (60.00) | 0 (0.00) | **3 (100.00)^e^** | 0 (0.00) |
| CG/cenAA/telBB | **1 (20.00)^f^** | 1 (50.00) | **0 (0.00)^g^** | 4 (100.00) |
| CC/cenAA/telBB | 1 (20.00) | 1 (50.00) | 0 (0.00) | 0 (0.00) |
|  | N = 125 | N = 72 | N = 42 | N = 78 |
| GG/cenAB/telAA | 67 (53.60) | 39 (54.17) | 21 (50.00) | 42 (53.85) |
| CG/cenAB/telAA | 53 (42.40) | 30 (41.67) | 21 (50.00) | 30 (38.46) |
| CC/cenAB/telAA | 5 (4.00) | 3 (4.16) | 0 (0.00) | 6 (7.69) |
|  |  |  |  |  |
|  | N = 102 | N = 60 | N = 36 | N = 76 |
| GG/cenAB/telAB | 55 (53.92) | 31 (51.67) | 21 (58.33) | 42 (55.26) |
| CG/cenAB/telAB | 44 (43.14) | 27 (45.00) | 14 (38.89) | 31 (40.79) |
| CC/cenAB/telAB | 3 (2.94) | 2 (3.33) | 1 (2.78) | 3 (3.95) |
|  | N = 17 | N = 9 | N = 4 | N = 11 |
| GG/cenAB/telBB | 7 (41.18) | 2 (22.22) | 3 (75.00) | 6 (54.55) |
| CG/cenAB/telBB | 9 (52.94) | 7 (77.78) | 1 (25.00) | 4 (36.36) |
| CC/cenAB/telBB | 1 (5.88) | 0 (0.00) | 0 (0.00) | 1 (9.09) |
|  | N = 24 | N = 15 | N = 5 | N = 16 |
| GG/cenBB/telAA | 19 (79.17) | 12 (80.00) | 4 (80.00) | 9 (56.25) |
| CG/cenBB/telAA | 5 (20.83) | 3 (20.00) | 1 (20.00) | 6 (37.50) |
| CC/cenBB/telAA | 0 (0.00) | 0 (0.00) | 0 (0.00) | 1 (6.25) |
|  | N = 20 | N = 10 | N = 7 | N = 22 |
| GG/cenBB/telAB | 13 (65.00) | **9 (90.00)^h^** | 3 (42.86) | 9 (40.91) |
| CG/cenBB/telAB | 7 (35.00) | **1 (10.00)^i^** | 4 (57.14) | 11 (50.00) |
| CC/cenBB/telAB | 0 (0.00) | 0 (0.00) | 0 (0.00) | 2 (9.09) |
|  | N = 11 | N = 5 | N = 6 | N = 8 |
| GG/cenBB/telBB | 6 (54.55) | 1 (20.00) | 5 (83.33) | 5 (62.50) |
| CG/cenBB/telBB | 5 (45.45) | 4 (80.00) | 1 (16.67) | 3 (37.50) |
| CC/cenBB/telBB | 0 (0.00) | 0 (0.00) | 0 (0.00) | 0 (0.00) |

IVF-ET – in vitro fertilization embryo transfer; RIF – recurrent implantation failure; SIVF – successful pregnancy after IVF-ET; p – probability; p_corr._ – probability after Bonferroni correction for multiple comparisons (x 6 for AA+/Bx combinations; x 9 for KIR centromeric or telomeric combinations; x 27 for KIR centromeric and telomeric combinations); OR – odds ratio; 95% CI – confidence interval from two-sided Fisher’s exact test; ns – not significant. Values in bold indicate significant differences. Values in parentheses are in percentages.

**SIVF vs. Fertile**: ^a^p/p_corr._ = 0.046/ns, OR = 0.160, 95% CI (0.00-1.08); ^c^p/p_corr._ = 0.039/ns, OR = 5.712, 95% CI (0.93-64.52); ^e^p/p_corr._ = 0.029/ns, OR = Inf., 95% CI (0.93-Inf.); ^g^p/p_corr._ = 0.029/ns, OR = 0.000, 95% CI (0.00-1.08);

**RIF vs. SIFV**: ^b^p/p_corr._ = 0.001/0.006, OR = 0.049, 95% CI (0.00-0.38); ^d^p/p_corr._ = 0.003/0.023, OR = 14.540, 95% CI (2.00-191.36);

**All IVF vs. Fertile:** ^f^p/p_corr._ = 0.048/ns, OR = 0.000, 95% CI (0.00-1.28);

**RIF vs. Fertile**: ^h^p/p_corr._ = 0.019/ns, OR = 12.030, 95% CI (1.28-610.25); ^i^p/p_corr._ = 0.050/ns, OR = 0.118, 95% CI (0.00-1.11)
