# Supplementary material for: ERAP, KIR, and HLA-C Profile in Recurrent Implantation Failure
Source: Front Immunol. 2021 Oct 22;12:755624. doi: 10.3389/fimmu.2021.755624 (PMC8569704; doi:10.3389/fimmu.2021.755624)
Supplement: Supplementary file 8 [file Table_8.docx]

**Supplementary Table 8** Association between ERAP1 rs27044 and KIR polymorphism in women participated in IVF-ET and fertile control.

| **ERAP1 rs27044/KIR** | **All IVF** | **RIF** | **SIVF** | **Fertile** |
| --- | --- | --- | --- | --- |
|  | N = 138 | N = 77 | N = 44 | N = 110 |
| CC/AA+ | 68 (49.28) | 37 (48.05) | 24 (54.55) | 62 (56.36) |
| CG/AA+ | 55 (39.86) | 29 (37.66) | 19 (43.18) | 37 (33.64) |
| GG/AA+ | 15 (10.86) | **11 (14.29)^a^** | 1 (2.27) | 11 (10.00) |
|  | N = 358 | N = 206 | N = 117 | N = 275 |
| CC/Bx+ | 196 (54.75) | 104 (50.49) | 64 (54.70) | 163 (59.27) |
| CG/Bx+ | 142 (39.66) | **91 (44.17)^b^** | 44 (37.61) | 96 (34.91) |
| GG/Bx+ | 20 (5.59) | 11 (5.34) | 9 (7.69) | 16 (5.82) |
|  | N = 197 | N = 112 | N = 61 | N = 173 |
| CC/cenAA | 96 (48.73) | 50 (44.64) | 33 (54.10) | 94 (54.34) |
| CG/cenAA | 81 (41.12) | 49 (43.75) | 24 (39.34) | 64 (36.99) |
| GG/cenAA | 20 (10.15) | 13 (11.61) | 4 (6.56) | 15 (8.67) |
|  | N = 244 | N = 141 | N = 82 | N = 166 |
| CC/cenAB | 132 (54.10) | **68 (48.23)^c^** | 47 (57.32) | 104 (62.65) |
| CG/cenAB | 98 (40.16) | **64 (45.39)^d^** | 30 (36.59) | 53 (31.93) |
| GG/cenAB | 14 (5.74) | 9 (6.38) | 5 (6.09) | 9 (5.42) |
|  | N = 55 | N = 30 | N = 18 | N = 46 |
| CC/cenBB | 36 (65.45) | **23 (76.67)^e^** | 8 (44.44) | 27 (58.70) |
| CG/cenBB | 18 (32.73) | 7 (23.33) | 9 (50.00) | 16 (34.78) |
| GG/cenBB | 1 (1.82) | 0 (0.00) | 1 (5.56) | 3 (6.52) |
|  | N = 286 | N = 164 | N = 90 | N = 205 |
| CC/telAA | 162 (56.64) | 92 (56.10) | 50 (55.56) | 122 (59.51) |
| CG/telAA | 103 (36.01) | 57 (34.76) | 37 (41.11) | 69 (33.66) |
| GG/telAA | 21 (7.35) | 15 (9.14) | 3 (3.33) | 14 (6.83) |
|  | N = 177 | N = 103 | N = 58 | N = 156 |
| CC/telAB | 85 (48.02) | **44 (42.72)^f^** | 30 (51.72) | 88 (56.41) |
| CG/telAB | 81 (45.76) | **53 (51.46)^g^** | 23 (39.66) | 58 (37.18) |
| GG/telAB | 11 (6.22) | 6 (5.82) | 5 (8.62) | 10 (6.41) |
|  | N = 33 | N = 16 | N = 13 | N = 23 |
| CC/telBB | 17 (51.52) | **5 (31.25)^h^** | 8 (61.54) | 15 (65.22) |
| CG/telBB | 13 (39.39) | **10 (62.50)^i^** | 3 (23.08) | 5 (21.74) |
| GG/telBB | 3 (9.09) | 1 (6.25) | 2 (15.38) | 3 (13.04) |
|  | N = 137 | N = 77 | N = 43 | N = 110 |
| CC/cenAA/telAA | 68 (49.64) | 37 (48.05) | 24 (55.81) | 62 (56.36) |
| CG/cenAA/telAA | 54 (39.42) | 29 (37.66) | 18 (41.86) | 37 (33.64) |
| GG/cenAA/telAA | 15 (10.94) | **11 (14.29)^j^** | 1 (2.33) | 11 (10.00) |
|  | N = 55 | N = 33 | N = 15 | N = 58 |
| CC/cenAA/telAB | 26 (47.27) | 13 (39.39) | 7 (46.67) | 30 (51.72) |
| CG/cenAA/telAB | 26 (47.27) | 19 (57.58) | 6 (40.00) | 26 (44.83) |
| GG/cenAA/telAB | 3 (5.46) | 1 (3.03) | 2 (13.33) | 2 (3.45) |
|  | N = 5 | N = 2 | N = 3 | N = 4 |
| CC/cenAA/telBB | 2 (40.00) | 0 (0.00) | 2 (66.67) | 2 (50.00) |
| CG/cenAA/telBB | 1 (20.00) | 1 (50.00) | 0 (0.00) | 0 (0.00) |
| GG/cenAA/telBB | 2 (40.00) | 1 (50.00) | 1 (33.33) | 2 (50.00) |
|  | N = 125 | N = 72 | N = 42 | N = 79 |
| CC/cenAB/telAA | 77 (61.60) | 45 (62.50) | 23 (54.76) | 52 (65.82) |
| CG/cenAB/telAA | 42 (33.60) | 23 (31.94) | 17 (40.48) | 25 (31.65) |
| GG/cenAB/telAA | 6 (4.80) | 4 (5.56) | 2 (4.76) | 2 (2.53) |
|  |  |  |  |  |
|  | N = 102 | N = 60 | N = 36 | N = 76 |
| CC/cenAB/telAB | 47 (46.08) | **22 (36.67)^k^** | 21 (58.33) | 44 (57.89) |
| CG/cenAB/telAB | 47 (46.08) | **33 (55.00)^l^** | 12 (33.33) | 26 (34.22) |
| GG/cenAB/telAB | 8 (7.84) | 5 (8.33) | 3 (8.34) | 6 (7.89) |
|  | N = 17 | N = 9 | N = 4 | N = 11 |
| CC/cenAB/telBB | 8 (47.06) | **1 (11.11)^m,n^** | 3 (75.00) | 8 (72.73) |
| CG/cenAB/telBB | 9 (52.94) | **8 (88.89)^o,p^** | 1 (25.00) | 2 (18.18) |
| GG/cenAB/telBB | 0 (0.00) | 0 (0.00) | 0 (0.00) | 1 (9.09) |
|  | N = 24 | N = 15 | N = 5 | N = 16 |
| CC/cenBB/telAA | 17 (70.83) | 10 (66.67) | 3 (60.00) | 8 (50.00) |
| CG/cenBB/telAA | 7 (29.17) | 5 (33.33) | 2 (40.00) | 7 (43.75) |
| GG/cenBB/telAA | 0 (0.00) | 0 (0.00) | 0 (0.00) | 1 (6.25) |
|  | N = 20 | N = 10 | N = 7 | N = 22 |
| CC/cenBB/telAB | 12 (60.00) | **9 (90.00)^q^** | 2 (28.57) | 14 (63.64) |
| CG/cenBB/telAB | 8 (40.00) | **1 (10.00)^r^** | 5 (71.43) | 6 (27.27) |
| GG/cenBB/telAB | 0 (0.00) | 0 (0.00) | 0 (0.00) | 2 (9.09) |
|  | N = 11 | N = 5 | N = 6 | N = 8 |
| CC/cenBB/telBB | 7 (63.64) | 4 (80.00) | 3 (50.00) | 5 (62.50) |
| CG/cenBB/telBB | 3 (27.27) | 1 (20.00) | 2 (33.33) | 3 (37.50) |
| GG/cenBB/telBB | 1 (9.09) | 0 (0.00) | 1 (16.67) | 0 (0.00) |

IVF-ET – in vitro fertilization embryo transfer; RIF – recurrent implantation failure; SIVF – successful pregnancy after IVF-ET; p – probability; p_corr._ – probability after Bonferroni correction for multiple comparisons (x 6 for AA+/Bx combinations; x 9 for KIR centromeric or telomeric combinations; x 27 for KIR centromeric and telomeric combiantions); OR – odds ratio; 95% CI – confidence interval from two-sided Fisher’s exact test; ns – not significant. Values in bold indicate significant differences. Values in parentheses are in percentages.

**RIF vs. SIVF:** ^a^p/p_corr._ = 0.054/ns, OR = 7.085, 95% CI (0.97-315.07); ^e^p/p_corr._ = 0.032/ns, OR = 3.974, 95% CI (0.99-17.38); ^j^p/p_corr._ = 0.054/ns, OR = 6.920, 95% CI (0.94-307.92); ^n^p/p_corr._ = 0.052/ns, OR = 0.062, 95% CI (0.00-1.46); ^p^p/p_corr._ = 0.052/ns, OR = 16.100, 95% CI (0.68-1388.84); ^q^p/p_corr._ = 0.035/ns, OR = 17.340, 95% CI (1.15-1165.33); ^r^p/p_corr._ = 0.035/ns, OR = 0.058, 95% CI (0.00-0.87);

**RIF vs. Fertile:** ^b^p/p_corr._ = 0.047/ns, OR = 1.474, 95% CI (1.00-2.17); ^c^p/p_corr._ = 0.015/ns, OR = 0.556, 95% CI (0.34-0.90); ^d^p/p_corr._ = 0.018/ns, OR = 1.769, 95% CI (1.08-2.90); ^f^p/p_corr._ = 0.042/ns, OR = 0.578, 95% CI (0.34-0.98); ^g^p/p_corr._ = 0.029/ns, OR = 1.787, 95% CI (1.05-3.06); ^h^p/p_corr._ = 0.054/ns, OR = 0.252, 95% CI (0.05-1.13); ^i^p/p_corr._ = 0.018/ns, OR = 5.689, 95% CI (1.20-31.53); ^k^p/p_corr._ = 0.016/ns, OR = 0.424, 95% CI (0.20-0.89); ^l^p/p_corr._ = 0.023/ns, OR = 2.335, 95% CI (1.11-5.00); ^m^p/p_corr._ = 0.010/ns, OR = 0.057, 95% CI (0.00-0.70); ^o^p/p_corr._ = 0.005/ns, OR = 27.326, 95% CI (2.06-1740.08)
