# Supplementary material for: ERAP, KIR, and HLA-C Profile in Recurrent Implantation Failure
Source: Front Immunol. 2021 Oct 22;12:755624. doi: 10.3389/fimmu.2021.755624 (PMC8569704; doi:10.3389/fimmu.2021.755624)
Supplement: Supplementary file 9 [file Table_9.docx]

**Supplementary Table 9** Association between ERAP1 rs30187 and KIR polymorphisms in women participated in IVF-ET and fertile control.

| **ERAP1 rs30187/KIR** | **All IVF** | **RIF** | **SIVF** | **Fertile** |
| --- | --- | --- | --- | --- |
|  | N = 138 | N = 77 | N = 44 | N = 110 |
| CC/AA+ | 54 (39.13) | 29 (37.66) | 19 (43.18) | 52 (47.27) |
| CT/AA+ | 63 (45.65) | 33 (42.86) | 22 (50.00) | 45 (40.91) |
| TT/AA+ | 21 (15.22) | 15 (19.48) | 3 (6.82) | 13 (11.82) |
|  | N = 358 | N = 206 | N = 117 | N = 275 |
| CC/Bx+ | 167 (46.65) | 89 (43.20) | 56 (47.86) | 133 (48.36) |
| CT/Bx+ | 156 (43.58) | 97 (47.09) | 48 (41.03) | 121 (44.00) |
| TT/Bx+ | 35 (9.77) | 20 (9.71) | 13 (11.11) | 21 (7.64) |
|  | N = 197 | N = 112 | N = 61 | N = 173 |
| CC/cenAA | 79 (40.10) | 40 (35.71) | 28 (45.90) | 78 (45.09) |
| CT/cenAA | 87 (44.16) | 51 (45.54) | 27 (44.26) | 77 (44.51) |
| TT/cenAA | 31 (15.74) | **21 (18.75)^a^** | 6 (9.84) | 18 (10.40) |
|  | N = 244 | N = 141 | N = 82 | N = 166 |
| CC/cenAB | 111 (45.49) | 59 (41.84) | 39 (47.56) | 86 (51.81) |
| CT/cenAB | 110 (45.08) | 69 (48.94) | 34 (41.46) | 68 (40.96) |
| TT/cenAB | 23 (9.43) | 13 (9.22) | 9 (10.98) | 12 (7.23) |
|  | N = 55 | N = 30 | N = 18 | N = 46 |
| CC/cenBB | 31 (56.36) | 19 (63.33) | 8 (44.44) | 21 (45.65) |
| CT/cenBB | 22 (40.00) | 10 (33.33) | 9 (50.00) | 21 (45.65) |
| TT/cenBB | 2 (3.64) | 1 (3.34) | 1 (5.56) | 4 (8.70) |
|  | N = 286 | N = 164 | N = 90 | N = 205 |
| CC/telAA | 129 (45.10) | 74 (45.12) | 39 (43.33) | 104 (50.73) |
| CT/telAA | 125 (43.71) | 68 (41.46) | 45 (50.00) | 84 (40.98) |
| TT/telAA | 32 (11.19) | 22 (13.42) | 6 (6.67) | 17 (8.29) |
|  | N = 177 | N = 103 | N = 58 | N = 156 |
| CC/telAB | 78 (44.07) | 41 (39.81) | 28 (48.28) | 68 (43.59) |
| CT/telAB | 81 (45.76) | 53 (51.46) | 22 (37.93) | 73 (46.79) |
| TT/telAB | 18 (10.17) | 9 (8.73) | 8 (13.79) | 15 (9.62) |
|  | N = 33 | N = 16 | N = 13 | N = 23 |
| CC/telBB | 14 (42.42) | **3 (18.75)^b,c^** | 8 (61.54) | 13 (56.52) |
| CT/telBB | 13 (39.39) | 9 (56.25) | 3 (23.08) | 8 (34.78) |
| TT/telBB | 6 (18.18) | 4 (25.00) | 2 (15.38) | 2 (8.70) |
|  | N = 137 | N = 77 | N = 43 | N = 110 |
| CC/cenAA/telAA | 54 (39.42) | 29 (37.66) | 19 (44.19) | 52 (47.27) |
| CT/cenAA/telAA | 62 (45.26) | 33 (42.86) | 21 (48.84) | 45 (40.91) |
| TT/cenAA/telAA | 21 (15.32) | 15 (19.48) | 3 (6.97) | 13 (11.82) |
|  | N = 55 | N = 33 | N = 15 | N = 58 |
| CC/cenAA/telAB | 23 (41.82) | 11 (33.33) | 7 (46.67) | 24 (41.38) |
| CT/cenAA/telAB | 24 (43.64) | 17 (51.52) | 6 (40.00) | 31 (53.45) |
| TT/cenAA/telAB | 8 (14.54) | 5 (15.15) | 2 (13.33) | 3 (5.17) |
|  | N = 5 | N = 2 | N = 3 | N = 4 |
| CC/cenAA/telBB | 2 (40.00) | 0 (0.00) | 2 (66.67) | 2 (50.00) |
| CT/cenAA/telBB | 1 (20.00) | 1 (50.00) | 0 (0.00) | 0 (0.00) |
| TT/cenAA/telBB | 2 (40.00) | 1 (50.00) | 1 (33.33) | 2 (50.00) |
|  | N = 125 | N = 72 | N = 42 | N = 79 |
| CC/cenAB/telAA | 61 (48.80) | 36 (50.00) | 18 (42.86) | 44 (55.70) |
| CT/cenAB/telAA | 53 (42.40) | 29 (40.28) | 21 (50.00) | 32 (40.50) |
| TT/cenAB/telAA | 11 (8.80) | 7 (9.72) | 3 (7.14) | 3 (3.80) |
|  |  |  |  |  |
|  | N = 102 | N = 60 | N = 36 | N = 76 |
| CC/cenAB/telAB | 43 (42.16) | 22 (36.67) | 18 (50.00) | 35 (46.05) |
| CT/cenAB/telAB | 49 (48.04) | **34 (56.67)^d^** | 12 (33.33) | 32 (42.11) |
| TT/cenAB/telAB | 10 (9.80) | 4 (6.66) | 6 (16.67) | 9 (11.84) |
|  | N = 17 | N = 9 | N = 4 | N = 11 |
| CC/cenAB/telBB | 7 (41.18) | **1 (11.11)^e,f^** | 3 (75.00) | 7 (63.64) |
| CT/cenAB/telBB | 8 (47.06) | 6 (66.67) | 1 (25.00) | 4 (36.36) |
| TT/cenAB/telBB | 2 (11.76) | 2 (22.22) | 0 (0.00) | 0 (0.00) |
|  | N = 24 | N = 15 | N = 5 | N = 16 |
| CC/cenBB/telAA | 14 (58.33) | 9 (60.00) | 2 (40.00) | 8 (50.00) |
| CT/cenBB/telAA | 10 (41.67) | 6 (40.00) | 3 (60.00) | 7 (43.75) |
| TT/cenBB/telAA | 0 (0.00) | 0 (0.00) | 0 (0.00) | 1 (6.25) |
|  | N = 20 | N = 10 | N = 7 | N = 22 |
| CC/cenBB/telAB | 12 (60.00) | 8 (80.00) | 3 (42.86) | 9 (40.91) |
| CT/cenBB/telAB | 8 (40.00) | 2 (20.00) | 4 (57.14) | 10 (45.45) |
| TT/cenBB/telAB | 0 (0.00) | 0 (0.00) | 0 (0.00) | 3 (13.64) |
|  | N = 11 | N = 5 | N = 6 | N = 8 |
| CC/cenBB/telBB | 5 (45.45) | 2 (40.00) | 3 (50.00) | 4 (50.00) |
| CT/cenBB/telBB | 4 (36.36) | 2 (40.00) | 2 (33.33) | 4 (50.00) |
| TT/cenBB/telBB | 2 (18.19) | 1 (20.00) | 1 (16.67) | 0 (0.00) |

IVF-ET – in vitro fertilization embryo transfer; RIF – recurrent implantation failure; SIVF – successful pregnancy after IVF-ET; p – probability; p_corr._ – probability after Bonferroni correction for multiple comparisons (x 6 for AA+/Bx combinations; x 9 for KIR centromeric or telomeric combinations; x 27 for KIR centromeric and telomeric combinations); OR – odds ratio; 95% CI – confidence interval from two-sided Fisher’s exact test; ns – not significant. Values in bold indicate significant differences. Values in parentheses are in percentages.

**RIF vs. Fertile**: ^a^p/p_corr._ = 0.053/ns, OR = 1.982, 95% CI (0.95-4.18); ^b^p/p_corr._ = 0.024/ns, OR = 0.186, 95% CI (0.03-0.94); ^e^p/p_corr._ = 0.028/ns, OR = 0.083, 95% CI (0.00-1.00);

**RIF vs. SIVF**: ^c^p/p_corr._ = 0.027/ns, OR = 0.156, 95% CI (0.02-0.98); ^d^p/p_corr._ = 0.035/ns, OR = 2.589, 95% CI (1.02-6.83); ^f^p/p_corr._ = 0.052/ns, OR = 0.062, 95% CI (0.00-1.46)
