# Supplementary material for: ERAP, KIR, and HLA-C Profile in Recurrent Implantation Failure
Source: Front Immunol. 2021 Oct 22;12:755624. doi: 10.3389/fimmu.2021.755624 (PMC8569704; doi:10.3389/fimmu.2021.755624)
Supplement: Supplementary file 10 [file Table_10.docx]

**Supplementary Table 10** Association between ERAP1 rs2287987 and KIR polymorphism in women participated in IVF-ET and fertile control.

| **ERAP1 rs2287987/KIR** | **All IVF** | **RIF** | **SIVF** | **Fertile** |
| --- | --- | --- | --- | --- |
|  | N = 138 | N = 77 | N = 44 | N = 110 |
| TT/AA+ | 89 (64.49) | 52 (67.53) | 27 (61.36) | 73 (66.36) |
| CT/AA+ | 46 (33.33) | 24 (31.17) | 15 (34.09) | 36 (32.73) |
| CC/AA+ | 3 (2.18) | 1 (1.30) | 2 (4.55) | 1 (0.91) |
|  | N = 358 | N = 206 | N = 117 | N = 275 |
| TT/Bx+ | 218 (60.89) | 123 (59.71) | 76 (64.96) | 155 (56.36) |
| CT/Bx+ | 119 (33.24) | 70 (33.98) | 35 (29.91) | 110 (40.00) |
| CC/Bx+ | 21 (5.87) | 13 (6.31) | 6 (5.13) | 10 (3.64) |
|  | N = 197 | N = 112 | N = 61 | N = 173 |
| TT/cenAA | 128 (64.97) | 74 (66.07) | 40 (65.57) | 111 (64.16) |
| CT/cenAA | 64 (32.49) | 35 (31.25) | 19 (31.15) | 59 (34.10) |
| CC/cenAA | 5 (2.54) | 3 (2.68) | 2 (3.28) | 3 (1.74) |
|  | N = 244 | N = 141 | N = 82 | N = 166 |
| TT/cenAB | 146 (59.84) | 84 (59.57) | 51 (62.20) | 91 (54.82) |
| CT/cenAB | 83 (34.02) | 48 (34.04) | 27 (32.93) | 68 (40.96) |
| CC/cenAB | 15 (6.14) | 9 (6.39) | 4 (4.87) | 7 (4.22) |
|  | N = 55 | N = 30 | N = 18 | N = 46 |
| TT/cenBB | 33 (60.00) | 17 (56.67) | 12 (66.67) | 26 (56.52) |
| CT/cenBB | 18 (32.73) | 11 (36.67) | 4 (22.22) | 19 (41.30) |
| CC/cenBB | 4 (7.27) | 2 (6.66) | 2 (11.11) | 1 (2.18) |
|  | N = 286 | N = 164 | N = 90 | N = 205 |
| TT/telAA | 172 (60.14) | 102 (62.20) | 54 (60.00) | 127 (61.95) |
| CT/telAA | 104 (36.36) | 56 (34.15) | 33 (36.67) | 72 (35.12) |
| CC/telAA | 10 (3.50) | 6 (3.65) | 3 (3.33) | 6 (2.93) |
|  | N = 177 | N = 103 | N = 58 | N = 156 |
| TT/telAB | 110 (62.15) | 60 (58.25) | 40 (68.97) | 90 (57.69) |
| CT/telAB | 57 (32.20) | 37 (35.92) | 15 (25.86) | 61 (39.10) |
| CC/telAB | 10 (5.65) | 6 (5.83) | 3 (5.17) | 5 (3.21) |
|  | N = 33 | N = 16 | N = 13 | N = 23 |
| TT/telBB | **25 (75.76)^a^** | **13 (81.25)^b^** | 9 (69.23) | 10 (43.48) |
| CT/telBB | **4 (12.12)^c^** | **1 (6.25)^d^** | **2 (15.38)^e^** | 13 (56.52) |
| CC/telBB | 4 (12.12) | 2 (12.50) | 2 (15.39) | 0 (0.00) |
|  | N = 137 | N = 77 | N = 43 | N = 110 |
| TT/cenAA/telAA | 88 (64.23) | 52 (67.53) | 26 (60.47) | 73 (66.36) |
| CT/cenAA/telAA | 46 (33.58) | 24 (31.17) | 15 (34.88) | 36 (32.73) |
| CC/cenAA/telAA | 3 (2.19) | 1 (1.30) | 2 (4.65) | 1 (0.91) |
|  | N = 55 | N = 33 | N = 15 | N = 58 |
| TT/cenAA/telAB | 35 (63.64) | 20 (60.61) | 11 (73.33) | 34 (58.62) |
| CT/cenAA/telAB | 18 (32.73) | 11 (33.33) | 4 (26.67) | 22 (37.93) |
| CC/cenAA/telAB | 2 (3.63) | 2 (6.06) | 0 (0.00) | 2 (3.45) |
|  | N = 5 | N = 2 | N = 3 | N = 4 |
| TT/cenAA/telBB | 5 (100.00) | 2 (100.00) | 3 (100.00) | 3 (75.00) |
| CT/cenAA/telBB | 0 (0.00) | 0 (0.00) | 0 (0.00) | 1 (25.00) |
| CC/cenAA/telBB | 0 (0.00) | 0 (0.00) | 0 (0.00) | 0 (0.00) |
|  | N = 125 | N = 72 | N = 42 | N = 79 |
| TT/cenAB/telAA | 71 (56.80) | 43 (59.72) | 24 (57.14) | 49 (62.03) |
| CT/cenAB/telAA | 48 (38.40) | 25 (34.72) | 17 (40.48) | 26 (32.91) |
| CC/cenAB/telAA | 6 (4.80) | 4 (5.56) | 1 (2.38) | 4 (5.06) |
|  |  |  |  |  |
|  | N = 102 | N = 60 | N = 36 | N = 76 |
| TT/cenAB/telAB | 63 (61.76) | 34 (56.67) | 25 (69.44) | 38 (50.00) |
| CT/cenAB/telAB | 32 (31.37) | 22 (36.67) | **9 (25.00)^f^** | 35 (46.05) |
| CC/cenAB/telAB | 7 (6.87) | 4 (6.66) | 2 (5.56) | 3 (3.95) |
|  | N = 17 | N = 9 | N = 4 | N = 11 |
| TT/cenAB/telBB | 12 (70.59) | 7 (77.78) | 2 (50.00) | 4 (36.36) |
| CT/cenAB/telBB | **3 (17.65)^g^** | **1 (11.11)^h^** | 1 (25.00) | 7 (63.64) |
| CC/cenAB/telBB | 2 (11.76) | 1 (11.11) | 1 (25.00) | 0 (0.00) |
|  | N = 24 | N = 15 | N = 5 | N = 16 |
| TT/cenBB/telAA | 13 (54.17) | 7 (46.67) | 4 (80.00) | 5 (31.25) |
| CT/cenBB/telAA | 10 (41.67) | 7 (46.67) | 1 (20.00) | 10 (62.50) |
| CC/cenBB/telAA | 1 (4.16) | 1 (6.66) | 0 (0.00) | 1 (6.25) |
|  | N = 20 | N = 10 | N = 7 | N = 22 |
| TT/cenBB/telAB | 12 (60.00) | 6 (60.00) | 4 (57.14) | 18 (81.82) |
| CT/cenBB/telAB | 7 (35.00) | 4 (40.00) | 2 (28.57) | 4 (18.18) |
| CC/cenBB/telAB | 1 (5.00) | 0 (0.00) | 1 (14.29) | 0 (0.00) |
|  | N = 11 | N = 5 | N = 6 | N = 8 |
| TT/cenBB/telBB | 8 (72.73) | 4 (80.00) | 4 (66.66) | 3 (37.50) |
| CT/cenBB/telBB | **1 (9.09)^i^** | 0 (0.00) | 1 (16.67) | 5 (62.50) |
| CC/cenBB/telBB | 2 (18.18) | 1 (20.00) | 1 (16.67) | 0 (0.00) |

IVF-ET – in vitro fertilization embryo transfer; RIF – recurrent implantation failure; SIVF – successful pregnancy after IVF-ET; p – probability; p_corr._ – probability after Bonferroni correction for multiple comparisons (x 6 for AA+/Bx combinations; x 9 for KIR centromeric or telomeric combinations; x 27 for KIR centromeric and telomeric combiantions); OR – odds ratio; 95% CI – confidence interval from two-sided Fisher’s exact test; ns – not significant. Values in bold indicate significant differences. Values in parentheses are in percentages.

**All IVF vs. Fertile:** ^a^p/p_corr_**_._** = 0.024/ns, OR = 3.952, 95% CI (1.12-14.98); ^c^p/p_corr._ = 0.001/0.007, OR = 0.111, 95% CI (0.02-0.46); ^g^p/p_corr._ = 0.020/ns, OR = 0.134, 95% CI (0.01-0.91); ^i^p/p_corr._ = 0.041/ns, OR = 0.072, 95% CI (0.00-0.98);

**RIF vs. Fertile:** ^b^p/p_corr._ = 0.024/ns, OR = 5.377, 95% CI (1.07-37.54); ^d^p/p_corr._ = 0.002/0.016, OR = 0.055, 95% CI (0.00-0.47); ^h^p/p_corr._ = 0.028/ns, OR = 0.083, 95% CI (0.00-1.00);

**SIVF vs. Fertile:** ^e^p/p_corr._ = 0.033/ns, OR = 0.148, 95% CI (0.01-0.91); ^f^p/p_corr._ = 0.039/ns, OR = 0.394, 95% CI (0.14-1.01)
