# Supplementary material for: ERAP, KIR, and HLA-C Profile in Recurrent Implantation Failure
Source: Front Immunol. 2021 Oct 22;12:755624. doi: 10.3389/fimmu.2021.755624 (PMC8569704; doi:10.3389/fimmu.2021.755624)
Supplement: Supplementary file 11 [file Table_11.docx]

**Supplementary Table 11** Association between ERAP1 rs6861666 and KIR polymorphism in women participated in IVF-ET and fertile control.

| **ERAP1 rs6861666/KIR** | **All IVF** | **RIF** | **SIVF** | **Fertile** |
| --- | --- | --- | --- | --- |
|  | N = 137 | N = 76 | N = 44 | N = 109 |
| AA/AA+ | 115 (83.94) | 65 (85.53) | 36 (81.82) | 96 (88.07) |
| AG/AA+ | 21 (15.33) | 10 (13.16) | 8 (18.18) | 13 (11.93) |
| GG/AA+ | 1 (0.73) | 1 (1.31) | 0 (0.00) | 0 (0.00) |
|  | N = 358 | N = 206 | N = 117 | N = 271 |
| AA/Bx+ | 309 (86.31) | 173 (83.98) | 102 (87.18) | 230 (84.87) |
| AG/Bx+ | 48 (13.41) | 33 (16.02) | 14 (11.97) | 40 (14.76) |
| GG/Bx+ | 1 (0.28) | 0 (0.00) | 1 (0.85) | 1 (0.37) |
|  | N = 196 | N = 111 | N = 61 | N = 170 |
| AA/cenAA | 167 (85.20) | 95 (85.59) | 51 (83.61) | 155 (91.18) |
| AG/cenAA | 28 (14.29) | 15 (13.51) | 10 (16.39) | 15 (8.82) |
| GG/cenAA | 1 (0.51) | 1 (0.90) | 0 (0.00) | 0 (0.00) |
|  | N = 244 | N = 141 | N = 82 | N = 164 |
| AA/cenAB | 205 (84.02) | 114 (80.85) | 71 (86.59) | 139 (84.76) |
| AG/cenAB | 38 (15.57) | 27 (19.15) | 10 (12.20) | 25 (15.24) |
| GG/cenAB | 1 (0.41) | 0 (0.00) | 1 (1.21) | 0 (0.00) |
|  | N = 55 | N = 30 | N = 18 | N = 46 |
| AA/cenBB | **52 (94.55)^a^** | **29 (96.67)^b^** | 16 (88.89) | 32 (69.57) |
| AG/cenBB | **3 (5.45)^c^** | **1 (3.33)^d^** | 2 (11.11) | 13 (28.26) |
| GG/cenBB | 0 (0.00) | 0 (0.00) | 0 (0.00) | 1 (2.17) |
|  | N = 285 | N = 163 | N = 90 | N = 203 |
| AA/telAA | 243 (85.26) | 139 (85.28) | 76 (84.44) | 177 (87.19) |
| AG/telAA | 41 (14.39) | 23 (14.11) | 14 (15.56) | 26 (12.81) |
| GG/telAA | 1 (0.35) | 1 (0.61) | 0 (0.00) | 0 (0.00) |
|  | N = 177 | N = 103 | N = 58 | N = 155 |
| AA/telAB | 152 (85.88) | 85 (82.52) | 51 (87.93) | 131 (84.52) |
| AG/telAB | 24 (13.56) | 18 (17.48) | 6 (10.34) | 23 (14.84) |
| GG/telAB | 1 (0.56) | 0 (0.00) | 1 (1.73) | 1 (0.64) |
|  | N = 33 | N = 16 | N = 13 | N = 22 |
| AA/telBB | 29 (87.88) | 14 (87.50) | 11 (84.62) | 18 (81.82) |
| AG/telBB | 4 (12.12) | 2 (12.50) | 2 (15.38) | 4 (18.18) |
| GG/telBB | 0 (0.00) | 0 (0.00) | 0 (0.00) | 0 (0.00) |
|  | N = 136 | N = 76 | N = 43 | N = 109 |
| AA/cenAA/telAA | 114 (83.82) | 65 (85.53) | 35 (81.40) | 96 (88.07) |
| AG/cenAA/telAA | 21 (15.44) | 10 (13.16) | 8 (18.60) | 13 (11.93) |
| GG/cenAA/telAA | 1 (0.74) | 1 (1.31) | 0 (0.00) | 0 (0.00) |
|  | N = 55 | N = 33 | N = 15 | N = 58 |
| AA/cenAA/telAB | 48 (87.27) | 28 (84.85) | 13 (86.67) | 56 (96.55) |
| AG/cenAA/telAB | 7 (12.73) | 5 (15.15) | 2 (13.33) | 2 (3.45) |
| GG/cenAA/telAB | 0 (0.00) | 0 (0.00) | 0 (0.00) | 0 (0.00) |
|  | N = 5 | N = 2 | N = 3 | N = 3 |
| AA/cenAA/telBB | 5 (100.00) | 2 (100.00) | 3 (100.00) | 3 (100.00) |
| AG/cenAA/telBB | 0 (0.00) | 0 (0.00) | 0 (0.00) | 0 (0.00) |
| GG/cenAA/telBB | 0 (0.00) | 0 (0.00) | 0 (0.00) | 0 (0.00) |
|  | N = 125 | N = 72 | N = 42 | N = 78 |
| AA/cenAB/telAA | 107 (85.60) | 60 (83.33) | 37 (88.10) | 70 (89.74) |
| AG/cenAB/telAA | 18 (14.40) | 12 (16.67) | 5 (11.90) | 8 (10.26) |
| GG/cenAB/telAA | 0 (0.00) | 0 (0.00) | 0 (0.00) | 0 (0.00) |
|  |  |  |  |  |
|  | N = 102 | N = 60 | N = 36 | N = 75 |
| AA/cenAB/telAB | 84 (82.35) | 47 (78.33) | 31 (86.11) | 60 (80.00) |
| AG/cenAB/telAB | 17 (16.67) | 13 (21.67) | 4 (11.11) | 15 (20.00) |
| GG/cenAB/telAB | 1 (0.98) | 0 (0.00) | 1 (2.78) | 0 (0.00) |
|  | N = 17 | N = 9 | N = 4 | N = 11 |
| AA/cenAB/telBB | 14 (82.35) | 7 (77.78) | 3 (75.00) | 9 (81.82) |
| AG/cenAB/telBB | 3 (17.65) | 2 (22.22) | 1 (25.00) | 2 (18.18) |
| GG/cenAB/telBB | 0 (0.00) | 0 (0.00) | 0 (0.00) | 0 (0.00) |
|  | N = 24 | N = 15 | N = 5 | N = 16 |
| AA/cenBB/telAA | 22 (91.67) | 14 (93.33) | 4 (80.00) | 11 (68.75) |
| AG/cenBB/telAA | 2 (8.33) | 1 (6.67) | 1 (20.00) | 5 (31.25) |
| GG/cenBB/telAA | 0 (0.00) | 0 (0.00) | 0 (0.00) | 0 (0.00) |
|  | N = 20 | N = 10 | N = 7 | N = 22 |
| AA/cenBB/telAB | **20 (100.00)^e^** | 10 (100.00) | 7 (100.00) | 15 (68.18) |
| AG/cenBB/telAB | **0 (0.00)^f^** | 0 (0.00) | 0 (0.00) | 6 (27.27) |
| GG/cenBB/telAB | 0 (0.00) | 0 (0.00) | 0 (0.00) | 1 (4.55) |
|  | N = 11 | N = 5 | N = 6 | N = 8 |
| AA/cenBB/telBB | 10 (90.91) | 5 (100.00) | 5 (83.33) | 6 (75.00) |
| AG/cenBB/telBB | 1 (9.09) | 0 (0.00) | 1 (16.67) | 2 (25.00) |
| GG/cenBB/telBB | 0 (0.00) | 0 (0.00) | 0 (0.00) | 0 (0.00) |

IVF-ET – in vitro fertilization embryo transfer; RIF – recurrent implantation failure; SIVF – successful pregnancy after IVF-ET; p – probability; p_corr._ – probability after Bonferroni correction for multiple comparisons (x 6 for AA+/Bx combinations; x 9 for KIR centromeric or telomeric combinations; x 27 for KIR centromeric and telomeric combinations); OR – odds ratio; 95% CI – confidence interval from two-sided Fisher’s exact test; ns – not significant. Values in bold indicate significant differences. Values in parentheses are in percentages.

**All IVF vs. Fertile:** ^a^p/p_corr._ = 0.001/0.010, OR = 7.435, 95% CI (1.87-43.47); ^c^p/p_corr._ = 0.002/0.020, OR = 0.149, 95% CI (0.03-0.60); ^e^p/p_corr._ = 0.009/ns, OR = Inf., 95% CI (1.58-Inf.); ^f^p/p_corr._ = 0.022/ns, OR = 0.000, 95% CI (0.00-0.81);

**RIF vs. Fertile:** ^b^p/p_corr._ = 0.003/0.029, OR = 12.373, 95% CI (1.68-553.07); ^d^p/p_corr._ = 0.006/ns, OR = 0.090, 95% CI (0.00-0.67)
