# Supplementary material for: ERAP, KIR, and HLA-C Profile in Recurrent Implantation Failure
Source: Front Immunol. 2021 Oct 22;12:755624. doi: 10.3389/fimmu.2021.755624 (PMC8569704; doi:10.3389/fimmu.2021.755624)
Supplement: Supplementary file 13 [file Table_13.docx]

**Supplementary Table 13** Association between ERAP2 rs2248374 and KIR polymorphism in women participated in IVF-ET and fertile control.

| **ERAP2 rs2248374/KIR** | **All IVF** | **RIF** | **SIVF** | **Fertile** |
| --- | --- | --- | --- | --- |
|  | N = 137 | N = 76 | N = 44 | N = 109 |
| AA/AA+ | 33 (24.09) | 19 (25.00) | 10 (22.73) | 30 (27.52) |
| AG/AA+ | 64 (46.72) | 36 (47.37) | 23 (52.27) | 53 (48.62) |
| GG/AA+ | 40 (29.19) | 21 (27.63) | 11 (25.00) | 26 (23.86) |
|  | N = 358 | N = 206 | N = 117 | N = 273 |
| AA/Bx+ | 93 (25.98) | 48 (23.30) | 38 (32.48) | 76 (27.84) |
| AG/Bx+ | 173 (48.32) | 98 (47.57) | 53 (45.30) | 128 (46.89) |
| GG/Bx+ | 92 (25.70) | 60 (29.13) | 26 (22.22) | 69 (25.27) |
|  | N = 196 | N = 111 | N = 61 | N = 171 |
| AA/cenAA | 48 (24.49) | 29 (26.13) | 14 (22.95) | 45 (26.32) |
| AG/cenAA | 94 (47.96) | 52 (46.85) | 31 (50.82) | 84 (49.12) |
| GG/cenAA | 54 (27.55) | 30 (27.02) | 16 (26.23) | 42 (24.56) |
|  | N = 244 | N = 141 | N = 82 | N = 165 |
| AA/cenAB | 65 (26.64) | **31 (21.99)^a^** | 30 (36.59) | 47 (28.48) |
| AG/cenAB | 116 (47.54) | 67 (47.51) | 37 (45.12) | 78 (47.27) |
| GG/cenAB | 63 (25.82) | 43 (30.50) | 15 (18.29) | 40 (24.25) |
|  | N = 55 | N = 30 | N = 18 | N = 46 |
| AA/cenBB | 13 (23.64) | 7 (23.33) | 4 (22.22) | 14 (30.43) |
| AG/cenBB | 27 (49.09) | 15 (50.00) | 8 (44.44) | 19 (41.30) |
| GG/cenBB | 15 (27.27) | 8 (26.67) | 6 (33.34) | 13 (28.27) |
|  | N = 285 | N = 163 | N = 90 | N = 203 |
| AA/telAA | 74 (25.96) | 41 (25.15) | 24 (26.67) | 61 (30.05) |
| AG/telAA | 132 (46.32) | 79 (48.47) | 41 (45.56) | 94 (46.31) |
| GG/telAA | 79 (27.72) | 43 (26.38) | 25 (27.77) | 48 (23.64) |
|  | N = 177 | N = 103 | N = 58 | N = 156 |
| AA/telAB | 46 (25.99) | 25 (24.27) | 20 (34.48) | 38 (24.36) |
| AG/telAB | 86 (48.59) | 45 (43.69) | 28 (48.28) | 77 (49.36) |
| GG/telAB | 45 (25.42) | **33 (32.04)^b^** | 10 (17.24) | 41 (26.28) |
|  | N = 33 | N = 16 | N = 13 | N = 23 |
| AA/telBB | 6 (18.18) | 1 (6.25) | 4 (30.77) | 7 (30.43) |
| AG/telBB | 19 (57.58) | 10 (62.50) | 7 (53.85) | 10 (43.48) |
| GG/telBB | 8 (24.24) | 5 (31.25) | 2 (15.38) | 6 (26.09) |
|  | N = 136 | N = 76 | N = 43 | N = 109 |
| AA/cenAA/telAA | 33 (24.26) | 19 (25.00) | 10 (23.26) | 30 (27.52) |
| AG/cenAA/telAA | 63 (46.32) | 36 (47.37) | 22 (51.16) | 53 (48.62) |
| GG/cenAA/telAA | 40 (29.42) | 21 (27.63) | 11 (25.58) | 26 (23.86) |
|  | N = 55 | N = 33 | N = 15 | N = 58 |
| AA/cenAA/telAB | 14 (25.45) | 10 (30.30) | 3 (20.00) | 14 (24.14) |
| AG/cenAA/telAB | 29 (52.73) | 15 (45.45) | 8 (53.33) | 30 (51.72) |
| GG/cenAA/telAB | 12 (21.82) | 8 (24.25) | 4 (26.67) | 14 (24.14) |
|  | N = 5 | N = 2 | N = 3 | N = 4 |
| AA/cenAA/telBB | 1 (20.00) | 0 (0.00) | 1 (33.33) | 1 (25.00) |
| AG/cenAA/telBB | 2 (40.00) | 1 (50.00) | 1 (33.33) | 1 (25.00) |
| GG/cenAA/telBB | 2 (40.00) | 1 (50.00) | 1 (33.33) | 2 (50.00) |
|  | N = 125 | N = 72 | N = 42 | N = 78 |
| AA/cenAB/telAA | 33 (26.40) | 17 (23.61) | 13 (30.95) | 27 (34.62) |
| AG/cenAB/telAA | 60 (48.00) | 36 (50.00) | 18 (42.86) | 34 (43.59) |
| GG/cenAB/telAA | 32 (25.60) | 19 (26.39) | 11 (26.19) | 17 (21.79) |
|  |  |  |  |  |
|  | N = 102 | N = 60 | N = 36 | N = 76 |
| AA/cenAB/telAB | 29 (28.43) | **13 (21.67)^c^** | **16 (44.44)^d^** | 18 (23.68) |
| AG/cenAB/telAB | 46 (45.10) | 25 (41.66) | 17 (47.23) | 38 (50.00) |
| GG/cenAB/telAB | 27 (26.47) | **22 (36.67)^e^** | **3 (8.33)^f^** | 20 (26.32) |
|  | N = 17 | N = 9 | N = 4 | N = 11 |
| AA/cenAB/telBB | 3 (17.65) | 1 (11.11) | 1 (25.00) | 2 (18.18) |
| AG/cenAB/telBB | 10 (58.82) | 6 (66.67) | 2 (50.00) | 6 (54.55) |
| GG/cenAB/telBB | 4 (23.53) | 2 (22.22) | 1 (25.00) | 3 (27.27) |
|  | N = 24 | N = 15 | N = 5 | N = 16 |
| AA/cenBB/telAA | 8 (33.33) | 5 (33.33) | 1 (20.00) | 4 (25.00) |
| AG/cenBB/telAA | 9 (37.50) | 7 (46.67) | 1 (20.00) | 7 (43.75) |
| GG/cenBB/telAA | 7 (29.17) | 3 (20.00) | 3 (60.00) | 5 (31.25) |
|  | N = 20 | N = 10 | N = 7 | N = 22 |
| AA/cenBB/telAB | 3 (15.00) | 2 (20.00) | 1 (14.28) | 6 (27.27) |
| AG/cenBB/telAB | 11 (55.00) | 5 (50.00) | 3 (42.86) | 9 (40.91) |
| GG/cenBB/telAB | 6 (30.00) | 3 (30.00) | 3 (42.86) | 7 (31.82) |
|  | N = 11 | N = 5 | N = 6 | N = 8 |
| AA/cenBB/telBB | 2 (18.18) | 0 (0.00) | 2 (33.33) | 4 (50.00) |
| AG/cenBB/telBB | 7 (63.64) | 3 (60.00) | 4 (66.67) | 3 (37.50) |
| GG/cenBB/telBB | 2 (18.18) | 2 (40.00) | 0 (0.00) | 1 (12.50) |

IVF-ET – in vitro fertilization embryo transfer; RIF – recurrent implantation failure; SIVF – successful pregnancy after IVF-ET; p – probability; p_corr._ – probability after Bonferroni correction for multiple comparisons (x 6 for AA+/Bx combinations; x 9 for KIR centromeric or telomeric combinations; x 27 for KIR centromeric and telomeric combinations); OR – odds ratio; 95% CI – confidence interval from two-sided Fisher’s exact test; ns – not significant. Values in bold indicate significant differences. Values in parentheses are in percentages.

**RIF vs. SIVF:** ^a^p/p_corr._ = 0.020/ns, OR = 0.490, 95% CI (0.26-0.93); ^b^p/p_corr_ = 0.044/ns, OR = 2.252, 95% CI (0.97-5.62); ^c^p/p_corr._ = 0.023/ns, OR = 0.350, 95% CI (0.13-0.94); ^e^p/p_corr._ = 0.002/0.051, OR = 6.262, 95% CI (1.66-35.58);

**SIVF vs. Fertile:** ^d^p/p_corr._ = 0.030/ns, OR = 2.554, 95% CI (1.01-6.50); ^f^p/p_corr._ = 0.043/ns, OR = 0.257, 95% CI (0.05-0.97)
