# Supplementary material for: ERAP, KIR, and HLA-C Profile in Recurrent Implantation Failure
Source: Front Immunol. 2021 Oct 22;12:755624. doi: 10.3389/fimmu.2021.755624 (PMC8569704; doi:10.3389/fimmu.2021.755624)
Supplement: Supplementary file 14 [file Table_14.docx]

**Supplementary Table 14** Association between ERAP haplotype and HLA-C polymorphism in women participated in IVF-ET and fertile control.

| **ERAP haplotype/**  **HLA-C** | **All IVF** | **RIF** | **SIVF** | **Fertile** |
| --- | --- | --- | --- | --- |
|  | N = 341 | N = 168 | N = 135 | N = 274 |
| H1/C1+ | 197 (57.77) | 102 (60.71) | 76 (56.30) | 156 (56.93) |
| H1/C2+ | 144 (42.23) | 66 (39.29) | 59 (43.70) | 118 (43.07) |
|  | N = 224 | N = 115 | N = 85 | N = 182 |
| H1/C1C1 | 80 (35.71) | 49 (42.61) | 26 (30.59) | 64 (35.16) |
| H1/C1C2 | 117 (52.23) | 53 (46.09) | 50 (58.82) | 92 (50.55) |
| H1/C2C2 | 27 (12.06) | 13 (11.30) | 9 (10.59) | 26 (14.29) |
|  | N = 152 | N = 95 | N = 32 | N = 104 |
| H4/C1+ | 79 (51.97) | 52 (54.74) | 15 (46.88) | 59 (56.73) |
| H4/C2+ | 73 (48.03) | 43 (45.26) | 17 (53.12) | 45 (43.27) |
|  | N = 95 | N = 60 | N = 21 | N = 73 |
| H4/C1C1 | 22 (23.16) | 17 (28.33) | 4 (19.05) | 28 (38.36) |
| H4/C1C2 | 57 (60.00) | 35 (58.33) | 11 (52.38) | 31 (42.46) |
| H4/C2C2 | 16 (16.84) | 8 (13.34) | 6 (28.57) | 14 (19.18) |
|  | N = 35 | N = 18 | N = 8 | N = 12 |
| H16/C1+ | 17 (48.57) | 7 (38.89) | 5 (62.50) | 7 (58.33) |
| H16/C2+ | 18 (51.43) | 11 (61.11) | 3 (37.50) | 5 (41.67) |
|  | N = 23 | N = 12 | N = 6 | N = 8 |
| H16/C1C1 | 5 (21.74) | 1 (8.33) | 3 (50.00) | 3 (37.50) |
| H16/C1C2 | 12 (52.17) | 6 (50.00) | 2 (33.33) | 4 (50.00) |
| H16/C2C2 | 6 (26.09) | 5 (41.67) | 1 (16.67) | 1 (12.50) |

IVF-ET – in vitro fertilization embryo transfer; RIF – recurrent implantation failure; SIVF – successful pregnancy after IVF-ET; Values in parentheses are in percentages.
