# Supplementary material for: ERAP, KIR, and HLA-C Profile in Recurrent Implantation Failure
Source: Front Immunol. 2021 Oct 22;12:755624. doi: 10.3389/fimmu.2021.755624 (PMC8569704; doi:10.3389/fimmu.2021.755624)
Supplement: Supplementary file 15 [file Table_15.docx]

**Supplementary Table 15** Association between ERAP haplotype and KIR in women participated in IVF-ET and fertile control.

| **ERAP haplotype /**  **KIR genotype** | **All IVF** | **RIF** | **SIVF** | **Fertile** |
| --- | --- | --- | --- | --- |
|  | N = 224 | N = 115 | N = 85 | N = 184 |
| H1/AA+ | 53 (23.66) | 31 (26.96) | 17 (20.00) | 58 (31.52) |
| H1/Bx+ | 171 (76.34) | 84 (73.04) | 68 (80.00) | 126 (68.48) |
|  | N = 95 | N = 60 | N = 21 | N = 73 |
| H4/AA+ | 21 (22.11) | 13 (21.67) | 6 (28.57) | 12 (16.44) |
| H4/Bx+ | 74 77.89) | 47 (78.33) | 15 (71.43) | 61 (83.56) |
|  | N = 23 | N = 12 | N = 6 | N = 8 |
| H16/AA+ | 7 (30.43) | 3 (25.00) | 3 (50.00) | 3 (37.50) |
| H16/Bx+ | 16 (69.57) | 9 (75.00) | 3 (50.00) | 5 (62.50) |

IVF-ET – in vitro fertilization embryo transfer; RIF – recurrent implantation failure; SIVF – successful pregnancy after IVF-ET
